# A genome-wide association study identifies novel genetic variants associated with neck or shoulder pain in the UK biobank (N = 430,193)

**DOI:** 10.1097/PR9.0000000000001267

**Published:** 2025-04-18

**Authors:** Yiwen Tao, Qi Pan, Tengda Cai, Zen Huat Lu, Mainul Haque, Tania Dottorini, Lesley A. Colvin, Blair H. Smith, Weihua Meng

**Affiliations:** a Nottingham Ningbo China Beacons of Excellence Research and Innovation Institute, University of Nottingham Ningbo China, Ningbo, China; b PAPRSB Institute of Health Sciences, Universiti Brunei Darussalam, Bandar Seri Begawan, Brunei Darussalam; c School of Mathematical Sciences, University of Nottingham Ningbo China, Ningbo, China; d School of Veterinary Medicine and Science, University of Nottingham, Nottingham, United Kingdom; e Division of Population Health and Genomics, Ninewells Hospital and Medical School, University of Dundee, Dundee, United Kingdom; f Center for Public Health, Faculty of Medicine, Health and Life Sciences, School of Medicine, Dentistry and Biomedical Sciences, Queen's University Belfast, Belfast, United Kingdom

**Keywords:** Neck or shoulder pain, UK Biobank, Genetic correlations, Genome-wide association study, Genomics, Phenome-wide association analysis

## Abstract

**Introduction:**

Neck and shoulder pain are prevalent musculoskeletal disorders that significantly affect the quality of life for a substantial portion of the global population. Studies have shown that women are more susceptible than men.

**Objective:**

This study aims to discover genetic variants associated with neck or shoulder pain through a genome-wide association study (GWAS), using data from 430,193 participants in the UK Biobank.

**Methods:**

A genome-wide association study was performed adjusting for age, sex, BMI, and 8 population principal components. Significant and independent genetic variants were replicated by FinnGen.

**Results:**

The primary GWAS revealed 5 significant genetic loci (including 2 novel) associated with neck or shoulder pain, with the most significant single nucleotide polymorphism (SNP) being rs9889282 (*P* = 2.63 × 10^−12^) near *CA10* on chromosome 17. Two novel significant associations were detected on chromosomes 18 and 14, with the top SNPs being rs4608411 (*P* = 8.20 × 10^−9^) near *TCF4* and rs370565192 (*P* = 3.80 × 10^−8^) in *DCAF5*, respectively. Our secondary GWAS identified a single novel genetic locus in *SLC24A3* among males and 2 genetic loci (including one novel near *LINC02770*) among females. In the replication stage, the *SLC39A8* locus was weakly supported by the FinnGen cohort. The tissue expression analysis revealed a significant association between brain tissues and neck or shoulder pain.

**Conclusion:**

In summary, this study has identified novel genetic variants for neck or shoulder pain. Sex-stratified GWAS also suggested that sex played a role in the occurrence of the phenotype.

## Abstract 2

Supplemental Digital Content is Available in the Text.

Noverl genes for neck or shoulder pain were identified using new definition in the UK Biobank dataset and replicated in the FinnGen dataset.

## 1. Introduction

Musculoskeletal pain is a serious concern worldwide, being the second leading cause of disability and with the fourth highest impact on overall health.^55^ Neck and shoulder pain are the second and third most prevalent musculoskeletal diseases, which are major causes of disability.^9,34^ Given that the physical findings and symptoms of neck and shoulder discomfort are similar, these 2 types of pain are often discussed together.^10,33^ The characterization of neck and shoulder pain includes the appearance of stiffness, muscular tension, pressure, or pain in the areas stretching from the neck to the scapular arch.^21^

From a global perspective, the age-standardized median prevalence of neck pain and shoulder pain was 27.0 per 1,000 and 37.8 per 1,000, respectively.^24,30^ For shoulder problems alone, it has been reported that approximately half of all new episodes that present in medical practice resolve completely within 6 months, rising to 60% after a year.^57^ In addition, frequent recurring neck discomfort not only has a significant influence on an individual's daily living activities and quality of life, but it also leads to lost productivity and ongoing economic expenditure, resulting in a significant social burden.^23^

Several investigations have shown that the occurrence of neck or shoulder pain is significantly related to age, sex, body mass index, physical ability, psychological pressure, workplace, and whether suffering from diabetes.^16,18,20,21,41^ Mahmud et al. reported that 72% of women and 51% of men suffer from neck pain, indicating that women are more likely to be affected by neck pain than men.^31^ The prevalence of neck and shoulder pain in female adolescents also increases with age.^15^ In addition, compared to normal-weight women and men, obese women and men had a 19% and 22% greater risk of neck or shoulder pain, respectively.^36^

The importance of genetic factors in neck or shoulder pain has been demonstrated. Based on twin studies, environmental and genetic influences on the self-report of neck or shoulder pain have been found.^37^ It was shown that heritability accounted for 68% of the variance in susceptibility to nonspecific neck discomfort at the age of 11 to 12 years, whereas individual contextual factors accounted for 32% of the diversity in phenotype.^48^ In a previous genome-wide association study (GWAS) using the UK Biobank, the *FOXP2*, *LINC01572*, and *CA10* genes were identified to be significantly associated with neck or shoulder pain, with only *FOXP2* and *LINCO1572* being marginally replicated in another cohort.^33^

The purpose of the study was to identify novel genetic variants associated with neck or shoulder pain in the UK Biobank by conducting a GWAS on the phenotype using new definitions. Compared with our previous GWAS by Meng et al.,^33^ we used a new definition of controls, which generated a larger sample size. Replications of findings were tested by comparison with the FinnGen Biobank cohort.^27^ In addition, we performed new sex-specific GWAS to explore potential genetic variants specifically associated with males or females and post-GWAS analyses, which were not available at the time of the previous publication.

## 2. Methods

### 2.1. Information about cohorts

We obtained the cohorts from the UK Biobank. The case group was defined as people with neck or shoulder pain, and the control group was defined as people without neck or shoulder pain. Detailed data information is provided in supplemental digital content (http://links.lww.com/PR9/A299).

### 2.2. Design and replication of the genome-wide association study

In the primary GWAS, we aimed to understand the genetic basis of neck or shoulder pain. Recognizing the significance of sex in the manifestation of health conditions, our secondary analysis focused on conducting 2 sex-stratified separate GWAS. We employed the publicly accessible summary statistics of the knee arthrosis from the FinnGen dataset during the replication phase.^27^

### 2.3. Genome-wide association study and statistical analysis

We employed the fastGWA function in GCTA (v1.94.1) for GWAS association analysis, using a sparse genetic relationship matrix. Quality control steps excluded SNPs with INFO scores <0.3, minor allele frequencies <0.5%, and failed Hardy–Weinberg tests (*P* < 10^−6^). Adjustments were made for sex, age, BMI, and 8 population principal components. Data of white British genetic ancestry individuals in the UK Biobank were selected for this GWAS by R v4.2.2. A threshold of *P* < 5 × 10^−8^ was set for this GWAS (see Methods for details, supplemental digital content, http://links.lww.com/PR9/A299).

### 2.4. Genome-wide association study-associated analysis by functional mapping and annotation

Functional Mapping and Annotation (FUMA) was utilized to annotate GWAS results. Significant SNPs (*P* < 5 × 10^−8^) were mapped to genes using default FUMA parameters. Regional visualization was performed using Locus Zoom^42^ (see Methods, supplemental digital content, http://links.lww.com/PR9/A299).

### 2.5. Expression quantitative trait loci, chromatin interaction analysis, and positional mapping

We utilized expression quantitative trait loci (eQTL), chromatin interaction analysis, and positional mapping to explore the regulatory mechanisms of variants identified through GWAS.^56^ Cis-eQTL were examined for their impact on gene expression by interacting with variants within 1 Mb of the gene. Chromatin structure was analyzed to understand its role in gene regulation within the nucleus. Positional mapping was performed with a maximum allowable distance of 10 kb to integrate these analyses (see Methods, supplemental digital content, http://links.lww.com/PR9/A299).

### 2.6. Genetic correlation analysis by linkage disequilibrium score regression

Linkage Disequilibrium Score Regression (LDSC) was used to estimate genetic correlations and heritability, using the Complex-Traits Genetics Virtual Lab for additional genetic correlation calculations (see Methods, supplemental digital content, http://links.lww.com/PR9/A299).

### 2.7. Phenome-wide association analysis

Phenome-Wide Association Analysis (PheWAS) was conducted using GWAS ATLAS to explore associations between identified SNPs and other traits, only SNPs with associations whose *P* values were lower than 0.05 considered (see Methods, supplemental digital content, http://links.lww.com/PR9/A299).

## 3. Results

### 3.1. Description of the samples

In the initial phase of the UK Biobank study (2006–2010), a cohort of 501,708 participants completed a pain questionnaire. Of these participants, 101,305 reported experiencing “neck or shoulder pain” and were thus classified as “cases” for the study. In contrast, 340,452 participants, who did not report such pain, were classified as “controls.” After the selection of white-British genetic ancestry participants and the exclusion of samples failing to meet QC criteria, 98,652 cases (42,869 males and 55,783 females) were included, with 331,541 controls (153,827 males and 177,714 females) in the primary GWAS analysis. This GWAS included 11,165,459 SNPs in the primary analysis. The secondary phase of our study involved sex-stratified GWAS analyses. In this phase, after identical QC procedures, the female cohort (233,497 samples) consisted of 55,783 cases and 177,714 controls, while the male cohort (196,696 samples) included 42,869 cases and 153,827 controls. Table 1 presents a comprehensive overview of the clinical attributes of both the cases and control of the primary and secondary GWAS.

**Table 1:** Clinical characteristics of neck or shoulder pain cases and controls in the UK Biobank.

GWAS analysis	Covariates	Cases	Controls	*P*
Primary GWAS	Sex (male: female)	42,869:55,783	153,827:177,714	<0.001
	Age (y)	56.8 (7.95)	56.9 (8.01)	<0.001
	BMI (kg/m^3^)	27.9 (5.00)	27.3 (4.70)	<0.001
Male-specific GWAS	Age (y)	57.1 (8.06)	57.0 (8.09)	<0.05
	BMI (kg/m^3^)	28.3 (4.42)	27.7 (4.19)	<0.001
Female-specific GWAS	Age (y)	56.7 (7.85)	56.6 (7.94)	<0.05
	BMI (kg/m^3^)	27.6 (5.39)	26.9 (5.07)	<0.001

Total number of females: 233,497; total number of males: 196,696.

A $\chi^{2}$ test was used to test the difference of gender frequency between cases and controls, and an independent t test was used for other covariates.

Continuous covariates were presented as mean (standard deviation)

GWAS, genome-wide association study; BMI, body mass index.

### 3.2. Genome-wide association study and replication results

**Figure 1.**
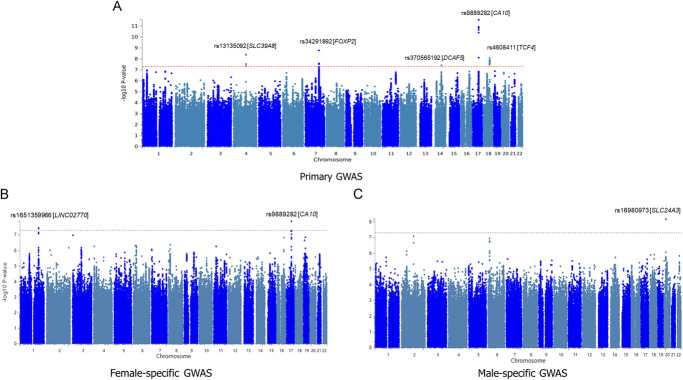
Manhattan plots of GWAS results for neck or shoulder pain in primary, female, and male analyses. Manhattan plots of the GWAS results for neck or shoulder pain across different analyses. Panel A shows the primary GWAS analysis for the overall samples (N = 430,193), panel B presents the female-specific analysis (N = 233,497), and panel C displays the male-specific analysis (N = 196,696). The *x*-axis represents chromosome positions across the 22 autosomes, and the *y*-axis shows the –log10 *P* values of SNP associations. The dotted line indicates the genome-wide significance threshold of 5 × 10^−8^.

In the primary GWAS, 5 GWAS signals were identified that demonstrated GWAS significant associations with neck or shoulder pain. These signals reached genome-wide significance with *P* values less than 5 × 10^−8^, as shown in Figure 1. Of these 5 loci, 2 are newly identified. Table 2 offers a comprehensive overview of the top SNPs within each locus. For a detailed list of all SNPs that displayed significant associations in this GWAS, please refer to supplemental digital content (see Table 1, http://links.lww.com/PR9/A300).

**Table 2:** The top SNPs within 5 loci identified by the GWAS on neck or shoulder pain.

Locus rank	rsID	Chr	SNP position	Nearest gene	UK biobank discovery stage					FinnGen replication		Identified or novel
					Effect allele	Noneffective allele	Frequency the effect allele	*P*	Beta	*P*	Beta	
1	rs9889282	17	50259142	*CA10*	A	C	0.61	2.63 × 10^–12^	−0.007	0.2007	0.0109	Meng et al.
2	rs34291892	7	114058731	*FOXP2*	CA	C	0.62	1.69 × 10^–9^	0.005	0.5052	0.2106	Meng et al.
3	rs13135092	4	103198082	*SLC39A8*	A	G	0.92	3.84 × 10^–9^	−0.01	0.0034	0.0914	Tsepilov et al.
4	rs4608411	18	52824637	*TCF4*	C	T	0.67	8.20 × 10^–9^	−0.005	0.9059	0.0010	Novel
5	rs370565192	14	69515858	*DCAF5*	TC	T	0.63	3.80 × 10^–8^	−0.005	0.2850	0.0169	Novel

GWAS, genome-wide association study; Chr, chromosome; SNP, single nucleotide polymorphism; *CA10*, carbonic anhydrase 10; *FOXP2*, Forkhead box protein P2; *SLC39A8*, solute carrier family 39 member 8; *TCF4*, Transcription factor 4; *DCAF5*, DDB1 and CUL4 associated factor 5.

**Figure 2.**
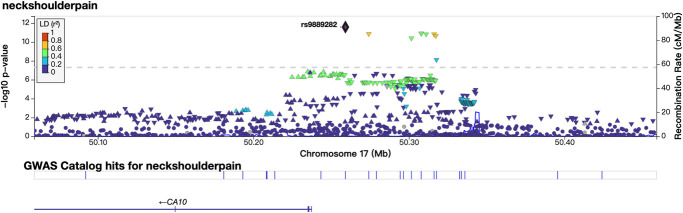
The regional plot of locus in the *CA10* region from the primary GWAS. Regional plot of the susceptibility locus in the *CA10* region based on the primary GWAS association analysis. The plot shows local SNP associations, with the lead SNP highlighted in purple. The *x*-axis represents the genomic position in megabases (Mb), while the *y*-axis displays the association strength of each SNP as –log10 *P* value.

**Figure 3.**
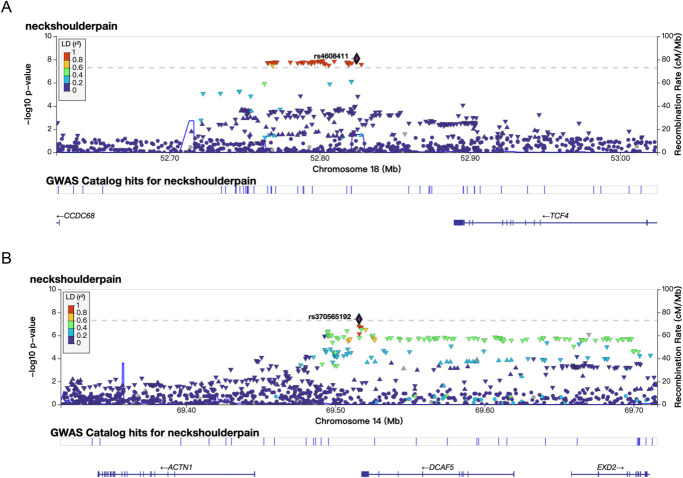
The regional plots of loci in the *TCF4* and *DCAF5* regions from the primary GWAS. Panel A shows the regional plot for the *TCF4* locus, and panel B displays the regional plot for the *DCAF5* locus. Each plot illustrates SNP associations in the respective regions from the primary GWAS analysis, with the lead SNP marked in purple. The *x*-axis represents the genomic position in Mb, while the *y*-axis displays the association strength of each SNP as –log10 *P* value.

The primary GWAS revealed the strongest association in the SNP cluster near the *CA10* gene on chromosome 17q21.33, with a *P* value of 2.63 × 10^−12^ for rs9889282. A significant association was also identified in the *FOXP2* gene on chromosome 7, with a lowest *P* value of 1.69 × 10^−9^ for rs34291892. Another notable association was identified in the *SLC39A8* gene on chromosome 4, with a lowest *P* value of 3.84 × 10^−9^ for rs13135092. In addition, significant associations were detected on chromosomes 18 and 14, with the top SNPs being rs4608411 (*P* = 8.20 × 10^−9^) near *TCF4* and rs370565192 (*P* = 3.80 × 10^−8^) in *DCAF5*, respectively. These loci on chromosomes 18 and 14 are reported for the first time in association with neck or shoulder pain. The regional plots displaying the most significant locus along with 2 novel loci are featured in Figures 2 and 3, while the regional plots for the additional 2 loci are available in supplemental digital content (see Figure 1, http://links.lww.com/PR9/A299). The Q–Q plot of the GWAS during the discovery phase is depicted in supplemental digital content (see Figure 2, http://links.lww.com/PR9/A299). The SNP-based heritability for neck or shoulder pain was determined to be 0.05 (*P* = 0.05 on the observed scale), with a standard error of 0.02. We extracted the *P* values for the associations of the 5 significant SNPs identified from a study on shoulder issues conducted by the FinnGen cohort. Among the 5 SNPs from the discovery cohort, rs13135092 in the *SLC39A8* gene was replicated weakly (*P* = 3.4 × 10^−3^) (Table 2).

In the secondary GWAS analyses, which were stratified by sex, the male-specific GWAS identified a single locus of genome-wide significance associated with neck or shoulder pain. This locus, distinct from those found in the primary GWAS, was located in the *SLC24A3* gene on chromosome 20, with a lowest *P* value of 6.52 × 10^−9^ for rs16980973. The female-specific GWAS revealed 2 significant loci associated with neck or shoulder pain (*CA10* and *LINC02770*), one of which differed from the primary GWAS findings. This novel locus was identified near the *LINC02770* gene on chromosome 1, with a *P* value of 3.57 × 10^−8^ for rs5779595. Detailed information on these findings is available in Table 3, and the corresponding Manhattan plots can be found in Figure 1.

**Table 3:** The top SNPs within 3 loci identified by the male-specific GWAS and female-specific GWAS on neck or shoulder pain.

Secondary GWAS	Locus rank	rsID	Chr	SNP position	Nearest gene	Effect allele	Noneffective allele	Frequency the effect allele	*P*	Beta
Male-specific GWAS	1	rs16980973	20	19648493	*SLC24A3*	A	T	0.87	6.52 × 10^−9^	0.012
Female-specific GWAS	1	rs9889282	17	50259142	*CA10*	A	C	0.61	1.31 × 10^−8^	−0.007
	2	rs5779595	1	191738609	*LINC02770*	CT	C	0.39	3.57 × 10^−8^	−0.007

GWAS, genome-wide association study; Chr, chromosome; SNP, single nucleotide polymorphism:*SLC24A3*, solute carrier family 24 member 3; *CA10*, carbonic anhydrase 10; *LINC02770*, long intergenic non-protein coding RNA 2770.

### 3.3. Gene, gene-set, and tissue expression analysis by functional mapping and annotation

In our gene analysis of primary GWAS, *FOXP2* displayed the most significant association. A total of 6 genes, namely *PABPC4, TCTA, MICB, FOXP2, IST1,* and *SLC44A2*, were found to be associated with neck or shoulder pain, each with a *P* value less than 2.60 × 10^−6^ (0.05/19,203). The detailed results of this analysis can be found in supplemental digital content (see Figure 3, http://links.lww.com/PR9/A300).

In the gene-set analysis, a comprehensive examination of 15,485 gene sets was conducted. Of these, one gene set, specifically “GOCC PRESYNAPTIC ENDOCYTIC ZONE,” was identified as significantly associated (*P* = 2.95 × 10^−7^). The top 10 gene sets from this analysis have been detailed in supplemental digital content (see Table 2, http://links.lww.com/PR9/A300).

**Figure 4.**
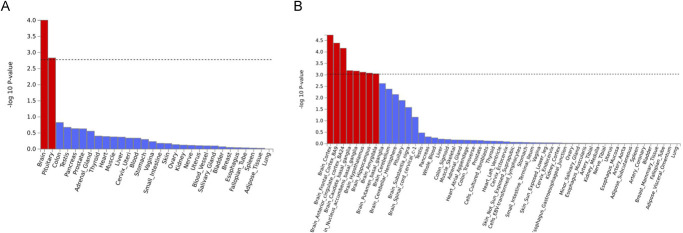
Tissue expression results from GTEx using FUMA. Panel A shows tissue expression results for 30 specific tissue types from GTEx, analyzed with FUMA. Panel B displays results for 53 tissue types. In both panels, the dashed line indicates the significance threshold, adjusted for multiple comparisons using Bonferroni correction. Significant tissue associations are highlighted in red.

In the tissue expression analysis, the broader categories of “Brain” and “Pituitary” showed statistical significance among 30 general tissue types across various organs. Notably, 8 brain-associated tissues, including “Brain Cortex,” “Brain Frontal Cortex BA9,” “Brain Anterior Cingulate Cortex BA24,” “Brain Caudate Basal Ganglia,” “Brain Nucleus Accumbens Basal Ganglia,” “Brain Hypothalamus,” “Brain Hippocampus,” and “Brain Amygdala” exhibited significant associations (*P* < 0.001) out of 53 specific tissue types from different organs. Further details and visualizations of these findings can be found in Figure 4.

In the gene expression heatmaps as shown in supplemental digital content (see Figure 4, http://links.lww.com/PR9/A299), genes *ACTN1*, *ERH*, *NARS*, *H2AFZ*, *UBE2D3*, *RDH2*, *RDH11*, and *VTL1B* demonstrated high expression across both all 53 tissue types and 30 tissue types, while genes *BDH2*, *COX11*, and *PIGH* were highly expressed in all tissue types except blood. The average of normalized expression allows comparison of gene expression across labels within a gene. Genes *C18orf54, SLC9B1, AC102948.2, KIF2B, STARD6, ASZ1,* and *C17ORF112* were most significantly expressed in testis. The expression of *CA10* was highest in brain tissues such as cerebellar hemisphere, putamen basal ganglia, and cortex.

### 3.4. Expression quantitative trait loci, chromatin interaction analysis, and positional mapping

**Figure 5.**
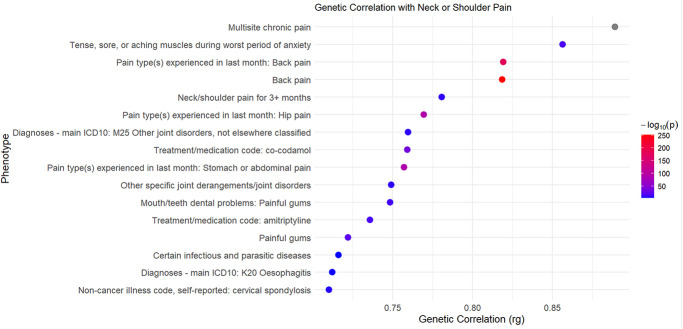
Genetic correlation results using LDSC on Complex-Traits Genetics Virtual Lab. This scatter plot illustrates the genetic correlations ($r_{g}$) between neck or shoulder pain and selected traits with $r_{g}$ values greater than 0.7, calculated using LDSC in the Complex-Traits Genetics Virtual Lab. The *x*-axis represents the genetic correlation coefficient ($r_{g}$), while the *y*-axis displays the phenotypes. Each dot indicates a trait, with its color gradient ranging from blue to red, where increasing redness reflects greater –log10 *P* values, indicating stronger statistical significance. For a full list of traits and their respective correlation values, see supplemental digital content (see Table 4, http://links.lww.com/PR9/A300).

The cis-eQTL analysis identified several significant SNPs associated with specific tissues, with notably low False Discovery Rates. Several SNPs in *FOXP2* on chromosome 7 were significantly associated with “Adipose_Subcutaneous tissue” and “Pituitary,” with *P* values of 1.02 × 10^−9^ and 1.98 × 10^−6^, and False Discovery Rates of 3.44 × 10^−8^ and 3.01 × 10^−14^, respectively. Complete data can be found in supplemental digital content (see Table 3, http://links.lww.com/PR9/A300). Chromatin interaction analysis uncovered significant gene-specific interactions. Genes related to *FOXP2* on chromosome 7 were *MET* and *MDFIC*, which were shown in supplemental digital content (see Figure 5, http://links.lww.com/PR9/A299). On chromosome 18, genes associated with *TCF4* included *NEDD4L*, *WDR7*, *TXNL1*, *STARD6*, *C18orf54*, and *RAB27B*. No significant genes were identified on chromosome 17. For *DCF4* on chromosome 14, the related genes were *GALNT16*, *EXD2*, and *ACTN1*. In addition, *SLC39A8* on chromosome 4 was linked to *UBE2D3*.

### 3.5. Genetic correlation analysis

In our analysis of the genetic correlations between neck or shoulder pain and other phenotypes by Complex-Traits Genetics Virtual Lab, several significant associations were identified. Neck or shoulder pain showed substantial genetic correlations with some other pain phenotypes, including multisite chronic pain ($r_{g}$ = 0.89, *P* = 0), back pain ($r_{g}$ = 0.82, *P* = 1.06 × 10^−175^), and hip pain ($r_{g}$ = 0.77, *P* = 9.26 × 10^−91^). In addition, significant positive genetic correlations were observed with neck or shoulder pain in relation to various medical conditions and health outcomes, including painful gums ($r_{g}$ = 0.72, *P* = 2.81 × 10^−23^), usage of amitriptyline ($r_{g}$ = 0.74, *P* = 1.26 × 10^−16^), and usage of co-codamol ($r_{g}$ = 0.76, *P* = 6.09 × 10^−38^). These results are comprehensively documented in supplemental digital content (see Table 4, http://links.lww.com/PR9/A300) and depicted in Figure 5. The genetic correlation for neck or shoulder pain between males and females was also calculated ($r_{g}$ = 0.79, *P* = 2.67 × 10^−41^).

### 3.6. Phenome-wide association analysis

Phenome-wide association analysis (PheWAS) was conducted using the GWAS ATLAS platform to investigate phenotypes associated with independent significantly associated SNPs (rs9889282, rs34291892, rs13135092, rs4608411, rs370565192, rs12951067) and significantly associated genes (*SLC39A8*, *FOXP2*, *DCAF5*, *CA10*, and *TCF4*). rs9889282 was significantly associated with the self-rated health trait (*P* = 3.82 × 10^−7^), while rs34291892 demonstrated a significant association with educational attainment (*P* = 1.70 × 10^−6^). Both rs13135092 and rs4608411 were associated with schizophrenia (*P* = 3.60 × 10^−12^ and *P* = 7.42 × 10^−5^, respectively). rs370565192 was associated with the Intelligence trait (*P* = 3.87 × 10^−5^), and rs12951067 was associated with the frequency of tiredness/lethargy in the last 2 weeks (*P* = 4.25 × 10^−5^). The genes *SLC39A8*, *FOXP2*, and *DCAF5* demonstrated associations with many traits after Bonferroni correction, covering a range of domains including ophthalmological, cellular, psychiatric, immunological, cardiovascular, and metabolic areas. The *CA10* and *TCF4* genes exhibited a strong association with psychiatric traits, specifically “morningness” (*P* = 5.42 × 10^−11^ for *CA10*) and schizophrenia (*P* = 3.64 × 10^−20^ for *TCF4*). All significant traits that passed Bonferroni correction are listed in supplemental digital content (see Table 5, http://links.lww.com/PR9/A300). The plots of phenotypes associated with SNPs and genes are visualized in supplemental digital content (see Figures 6 and 7, http://links.lww.com/PR9/A299), respectively.

For further interest, we also performed GWAS on acute neck or shoulder pain, sex-difference meta-analysis using GWAMA, Mendelian Randomization analysis, and 3-Way Venn diagram of sex-combined and sex-stratified GWAS credible sets. The results of these analyses are shown in the supplemental digital content (see Methods and Figures, http://links.lww.com/PR9/A299).

## 4. Discussion

These 3 GWAS on neck or shoulder pain utilizing the UK Biobank dataset identified several notable genetic loci associated with the phenotype, including 5 loci (2 novel) in the primary analysis, 2 loci in females, and one locus in males (Tables 2 and 3). In the replication stage, the *SLC39A8* locus was weakly supported by the FinnGen cohort.

In the primary GWAS, the locus near *CA10* on chromosome 17 was the most significantly associated with neck or shoulder pain, with a top SNP of rs9889282 (*P* = 2.63 × 10^−12^). *CA10* encodes a protein from the zinc metalloenzyme family called carbonic anhydrase, involved in bone resorption and bone mineral solubilization.^38,39^ The protein is also believed to be involved in the development of the brain and the central nervous system (https://www.ncbi.nlm.nih.gov/gene/105371829#gene-expression). The strong interconnections between carbonic anhydrase activity and neuropathic pain have been suggested.^3,4^ It has been identified that inhibition of carbonic anhydrase activity by intraperitoneal injection of acetazolamide decreased neuropathic pain.^7^ For *CA10*, one study suggested that it may play the role of adaptors, facilitating neurexins' indirect connections with unidentified postsynaptic target molecules, and thus mediating the development of new transsynaptic complexes.^51^ As there are not many studies on *CA10* in humans, future studies should focus on elucidating the mechanism of *CA10* action on neck or shoulder pain.

The second most significantly associated locus was identified in the *FOXP2* gene located on chromosome 7, with a top SNP of rs34291892 (*P* = 1.69 × 10^−9^). *FOXP2* is one member of the forkhead family of transcription factors and is located on human chromosome 7q31, which encodes a 715-amino acid protein 8. Alternatively, genes *DLX5* and *SYT4*, which are affected by *FOXP2*, are essential for brain development and function.^33^ In a previous GWAS analysis, *FOXP2* has been confirmed to be associated with neck or shoulder pain and multisite chronic pain.^22,33^ This discovery raises issues concerning the psychological factors that affect the onset and persistence of neck or shoulder pain.

The third notable association was identified in the *SLC39A8* gene on chromosome 4. *SLC39A8* encodes transporter zinc- and iron-related protein 8 (ZIP8).^13^ Genome-wide association study have revealed that human *SLC39A8*-deficient variants exhibit distinct multiorientation defects associated with clinical diseases in almost all organs including many musculoskeletal system.^35^ In the details of the central nervous system, target validation tests demonstrated that microRNA 488 can target *SLC39A8* mRNA, and inhibiting *SLC39A8* expression in the osteoarthritis animal model reduced cartilage deterioration.^47^ A subsequent paper has suggested that the increased expression of *SLC39A8* is associated with increased intracellular zinc levels in diseased chondrocytes among all zinc transporters in human and mouse cartilage affected by osteoarthritis.^26^ Musculoskeletal conditions, including osteoarthritis, can contribute to pain and discomfort in the neck and shoulder regions, making *SLC39A8* a potential player in these processes. It is worth noting that this locus near *SLC39A8* has already been found to be associated with chronic neck or shoulder pain in a GWAS analysis of chronic musculoskeletal pain.^54^

The 2 novel loci are near *TCF4* on chromosome 18 with a top SNP of rs4608411 (*P* = 8.20 × 10^−9^) and in *DCAF5* on chromosome 14 with a top SNP of rs370565192 (*P* = 3.80 × 10^−8^), respectively. *TCF4* encodes transcription factor 4, a fundamental helix-loop-helix transcription factor. The encoded protein is a member of the ubiquitous E-protein family, causes severe central nervous system and autonomous nervous system dysfunction when mutated in humans.^1^ Mutations in *TCF4* have been linked to a number of other psychiatric disorders including autism, bipolar disorder, and schizophrenia.^11,17,49,50^ The *DCAF5* gene is one part of the ubiquitin ligase complex *DDB1-CUL4*, and the *DCAF5* gene's brain expression results in *DDB1*- and *CUL4*-related factor 5.^2,29,45^ *CUL4B* makes up most of this complex, and mutations in it can result in X-linked intellectual impairment.^52^ *DCAF5* carries rare segregating variants in at least 2 independent families with bipolar disorder, which are hypothesized to be associated with psychiatric disorders.^12^ The discovery of these 2 new loci represents a significant further step forward in unraveling the complex genetic architecture of neck or shoulder pain.

In the sex-stratified GWAS analyses, male-specific GWAS revealed a locus n *SLC24A3* on chromosome 20, with a top SNP of rs16980973 (*P* = 6.52 × 10^−9^). *SLC24A3* encodes NCKX3, a potassium-dependent Na^+^/K^+^/Ca^2+^ exchanger that regulates intracellular calcium homeostasis.^6^ In the central nervous system, dysregulation of Ca^2+^ homeostasis can cause excitotoxicity and neurodegeneration.^53^ In the female-specific GWAS analysis, the most significant variant was identified near *CA10* same as the primary GWAS. The other significant variant is near the *LINC02770* gene on chromosome 1 with the top SNP rs5779595 (*P* = 3.57 × 10^−8^). Several GWAS have found that *LINC02770* is associated with insomnia and post-traumatic stress disorder (https://www.genecards.org/cg-ibin/carddisp.pl?gene=LINC02770). Although the specific genetic mechanism of *LINC02770* has not been explored, it introduces a novel aspect to the understanding of genetic factors contributing to pain susceptibility in females. The genetic correlation for neck or shoulder pain between males and females was also calculated ($r_{g}$ = 0.79), which suggests that the genetic mechanisms of neck or shoulder pain are not exactly the same between males and females. The reason for the difference is likely due to the important role of sex differences in the heterogeneity of pain.^14,46^

In the tissue expression analysis, neck or shoulder pain showed a strong correlation with the brain and pituitary categories. The identified brain tissues revealed key signaling pathways related to pain perception (BA24, hypothalamus, amygdala), sensory processing (amygdala, cortex, BA9, basal ganglia), and central neuroendocrine (hypothalamus).^19,25,28,32,40^ These findings align with clinical observations where individuals experiencing chronic neck or shoulder pain often report associated symptoms such as headaches, fatigue, and alterations in mood.^5,43^ The identified brain regions, including the cerebral cortex and amygdala, are known to play crucial roles in emotional processing and stress response.^44^ This connection raises questions about the psychosocial aspects influencing the development and persistence of neck or shoulder pain.

The genetic correlations between neck or shoulder pain and several other pain phenotypes suggest that these disorders share common genetic determinants. The 3 most highly correlated phenotypes of neck or shoulder pain were multisite chronic pain ($r_{g}$ = 0.89), back pain ($r_{g}$ = 0.82), and hip pain ($r_{g}$ = 0.77). The correlation between neck or shoulder pain and receipt of amitriptyline ($r_{g}$ = 0.74) and co-codamol ($r_{g}$ = 0.76) suggests that biological links exist between the neck or shoulder pain and each phenotype as amitriptyline is an antidepression drug although not used as a first-line treatment for depression due to considerable side effects and co-codamol is a pain-relief medicine. In the PheWAS, the results demonstrate that the top SNPs and corresponding genes are primarily highly associated with numerous psychiatric traits. This may provide further evidence that psychiatric factors could be an important contributor to neck or shoulder pain and/or vice versa. Meanwhile, these newly identified SNPs and genes are likely to play a role in the biological mechanism of neck or shoulder pain, which will have a suggestive effect on the development of targeted therapies (such as carbonic anhydrase inhibitors) for the pain.

Further to the previous GWAS on neck or shoulder pain by Meng et al.,^33^ this study has discovered 2 new loci. The added value of this study lies in its larger sample size, which is nearly double that of the previous study, enabled by a refined definition of controls that allowed for the inclusion of more data. The sex-stratified analyses and more comprehensive post-GWAS analyses including genetic correlation using LDSC and PheWAS have offered a broader understanding of the genetic architecture and phenotypic implications of the identified loci. In this study, we specifically examined the 5 significant SNPs identified in our discovery analysis and assessed their associations with FinnGen shoulder phenotypes.^27^ Among these, the SNP rs13135092 in the *SLC39A8* gene showed nominal replication with a *P* value of 0.0034, suggesting a weak but consistent association across independent populations. This approach differs from Meng et al. where replication was performed in 2 UK-based cohorts—GS:SFHS and TwinsUK—followed by a meta-analysis across these samples.^33^ By expanding our replication efforts to the FinnGen cohort, which is a more geographically diverse population, our study underscores the potential cross-population relevance of some of these loci.

It is important to note that cases and controls in the UK Biobank are self-reported and may be subject to several biases such as social expectations, recall periods, sampling methods, or selective recall. Therefore, these potential effects need to be carefully considered when interpreting the findings. We described cases and controls with neck or shoulder pain based on responses to specific questions from UK Biobank participants. However, this question was not designed to cover more detailed information about the severity and frequency of pain. Therefore, the phenotypic definitions we derived from these responses should be viewed as generalized. In addition, another limitation is particularly notable as we had to use a different phenotype (shoulder issues) instead of neck or shoulder pain. This substitution may have introduced a bias, as neck or shoulder pain, while a prominent indicator, is not entirely synonymous with shoulder issues. In the future, we plan to undertake more related studies such as MiXeR analyses, polygenic risk scores, and epidemiological odd ratios between neck or shoulder pain and other traits, to extend and deepen the insights gained from our current research.

## 5. Conclusion

In summary, our primary GWAS identified 5 genetic loci including 2 novel ones associated with neck or shoulder pain among all people with neck or shoulder pain. Our secondary GWAS identified a single novel genetic locus associated with neck or shoulder pain among males and 2 genetic loci (including one novel) associated with neck or shoulder pain among females. These discoveries not only expand our understanding of the genetic underpinnings of neck or shoulder pain but also emphasize the importance of considering sex-specific influences.

## Disclosures

The authors have no conflict of interest to declare.

## Appendix A. Supplemental digital content

Supplemental digital content associated with this article can be found online at http://links.lww.com/PR9/A299, http://links.lww.com/PR9/A300.
