# Supplementary material for: A genome-wide association study identifies novel genetic variants associated with neck or shoulder pain in the UK biobank (N = 430,193)
Source: Pain Rep. 2025 Apr 18;10(3):e1267. doi: 10.1097/PR9.0000000000001267 (PMC12026381; doi:10.1097/PR9.0000000000001267)
Supplement: Supplementary file 1 [file painreports-10-e1267-s001.pdf]

Supplementary Methods

Information about cohorts

The UK BioBank is a unique cohort, facilitating research into health and disease. The cohort is a large-scale collection of broad environmental, lifestyle and genetic data from more than 500,000 volunteers aged 40 to 69 years in the UK from 2006 to 2010. The volunteers gave informed consent to complete detailed questionnaires and provide biological samples including urine, saliva and blood (available at www.ukbiobank.ac.uk). Ethical approval for the study was obtained from the National Health Service and the National Research Ethics Service (reference 11/NW/0382). The genetic data and related data of half a million people in the UK are available for approved research projects into a wide range of diseases.

DNA extraction and quality control (QC) were standardized prior to the release of all data to the UK Biobank, and is described at https://biobank.ctsu.ox.ac.uk/crystal/ukb/docs/genotyping_sample_workflow.pdf. The Wellcome Trust Centre for Human Genetics at Oxford University is responsible for ensuring the reliability of genotyping results. QC steps include identifying poorly performing markers, assessing sample correlations, and accounting for batch effects. These detailed QC steps are available at http://biobank.ctsu.ox.ac.uk/crystal/refer.cgi?id=155580.

Case and control definitions

A questionnaire was designed by the UK Biobank including a specific pain-related question used in this study: "in the previous month, have you encountered any of the following that

interfered with your regular activities?" The alternatives were as follows: (1) Headache; (2) Facial pain; (3) Neck or shoulder pain; (4) Back pain; (5) Stomach or abdominal pain; (6) Hip pain; (7) Knee pain; (8) Pain all over the body; (9) None of the above; and (10) Prefer not to say (UK Biobank Questionnaire field ID: 6159). It is possible for volunteers to choose one or more answers. In this study, the cases of neck or shoulder pain were those who answered "yes" to "Neck or shoulder pain". The control group was defined by those who did not choose both "Neck or shoulder pain" and "Prefer not to say". In particular, this study used data from white British descent (field ID: 21000) in order to narrow the population stratification and make the results more specific and interpretable.

Design of the GWAS

The FinnGen dataset included 43,732 cases and 429,949 controls. The GWAS for the FinnGen cohort was conducted according to the protocols outlined in FinnGen GWAS round 9, detailed at https://finngen.gitbook.io/documentation/. Detailed information on sample phenotyping, genotyping, and GWAS methodology for the FinnGen sample can be reviewed at https://risteys.finngen.fi/.

GWAS and statistical analysis

Genome-wide complex trait analysis (GCTA, v1.94.1) is a statistical software for estimating the genetic contribution of complex traits across the genome, and is described at https://yanglab.westlake.edu.cn/software/gcta/#Overview [9]. Utilizing the fastGWA function within GCTA, a mixed linear model association tool was performed for GWAS association analysis by using a sparse genetic relationship matrix. The QC step involved excluding

single nucleotide polymorphisms (SNPs) with INFO scores below 0.3, minor allele frequencies lower than 0.5%, and failed Hardy-Weinberg tests ($p < 1 \times 10^{-6}$). In addition, mitochondrial SNPs and SNPs on the sex chromosomes were excluded. The association tests were adjusted for sex, age, BMI, and eight population principal components using the fastGWAS function in the primary GWAS. Similarly, the association tests were performed with adjustments for age, BMI, and eight population principal components in the secondary GWAS. R v4.2.2 was used to select the data of white British descent in UK Biobank data and divide the cases and controls of neck or shoulder pain. The difference in sex frequency between cases and controls was assessed by a x^2 test and other covariates were tested by independent t-tests using R v4.2.2, with significance determined by a p value threshold of 0.05. A threshold of $p < 5 \times 10^{-8}$ is generally considered to be significant for GWAS, which was applied for this study. Furthermore, GCTA was employed to calculate the narrow-sense heritability.

GWAS-associated analysis by FUMA

The functional mapping and annotation (FUMA) of GWAS web application is an annotation tool for gene analysis, gene-set analysis and tissue expression analysis of GWAS results [8]. The SNP2GENE function accepts as input GWAS summary results and gives thorough functional annotation for all SNPs in genomic regions highlighted by lead SNPs. The number of related loci is ascertained using the "maximum distance of linkage disequilibrium blocks to merge" method, while the number of significant independent SNPs is determined using the default r^2 value ($r^2 > 0.6$). In detail, the default FUMA parameters were utilized including the threshold of significant SNPs ($p < 5 \times 10^{-8}$) and the maximum distance of

linkage disequilibrium ($r^2 > 0.6$) with a minimum minor allele frequency (MAF) > 0.01. Also, the maximum distance between linkage disequilibrium blocks that might merge into a locus was set to less than 250 kb, and the 1000G Phase3 EUR population was utilized as the reference panel population. Furthermore, Locus Zoom (http://locuszoom.org/) was used to provide regional visualization [4].

The three key aspects of analysis facilitated by FUMA include gene-based association analysis and gene-set analysis conducted by integrating MAGMA (v1.0619) and tissue expression analysis generated by GTEx. In the aspect of gene analysis, the summary statistics of SNPs were integrated at the level of whole genes. Statistical information about the associated SNPs is aggregated at each locus to obtain information about the association with the studied phenotype at the gene level. Integration of the statistical information of SNPs presents the degree of association between each gene and the phenotype under study. All SNPs found inside genes were specifically mapped to 19,023 protein-coding genes and the definition of the significance was $p = 0.05/19{,}023 = 2.60 \times 10^{-6}$. In the gene-set analysis, FUMA performs collective analysis of genomes that share common biological, functional, or other features and the definition of the significance was set at $p = 0.05/15{,}485 = 3.23 \times 10^{-6}$. This approach helps to identify specific biological pathways, cellular functions, or other functional groups that further explain the genetic basis of the observed phenotypes. In the tissue expression analysis, FUMA uses tissue-specific expression data from GTEx to analyze the expression level of the specific genes in different tissues. We constructed four gene expression heatmaps for genes identified through positional mapping, incorporating average and normalized mean expression

values per label. The data used in these heatmaps was sourced from the GTEx v8 datasets, specifically 54 tissue types and 30 general tissue types. The tissue specificity was assessed utilizing differentially expressed genes (DEGs) predefined for each label from each expression dataset. Enrichment evaluations for DEGs were conducted for both tissue types from GTEx v8, targeting specifically positional mapping genes' enrichment without including the total distribution of SNP *p* values from the MAGMA tissue expression analysis.

Expression quantitative trait loci (eQTL), chromatin interaction analysis and positional mapping

Expression quantitative trait loci (eQTL) have become essential tools for understanding the regulatory mechanisms of variants identified through GWAS [6]. Specifically, cis-eQTL affect gene expression by interacting with variants located within 1 Mb of the gene. This proximity allows for a direct influence on gene regulation. In eukaryotic cells, the genome is densely packed within the small confines of the nucleus, with chromatin acting as the fundamental building block. This structural organization is essential for creating a three-dimensional configuration of the genome, which plays a critical role in processes such as DNA replication, repair of DNA damage, gene transcription, and other key biological functions [2]. In our studies, positional mapping was performed with a maximum allowable distance of 10 kb. This method combines cis-eQTL analysis, chromatin interaction analysis, and positional mapping to offer a thorough perspective on the genomic framework.

Genetic correlation analysis by LDSC

Linkage disequilibrium score regression (LDSC) is a statistical method used to estimate heritability and genetic associations from GWAS summary statistics, which is described at https://github.com/bulik/ldsc [1]. LDSC reveals the association between different phenotypes by quantifying the linkage disequilibrium between pairs of loci. In addition, LDSC can provide a genome-wide map of genetic correlation, showing the level of genetic correlation of different loci pairs, which helps to identify clusters of loci that are associated with the disease or phenotype as a whole. In this study, LDSC was also used to analyze the genetic correlation of neck or shoulder pain between males and females. The analysis was intended to reveal overall genetic difference between males and females for this phenotype. We also calculated genetic connections of neck or shoulder discomfort using 1,396 characteristics from the UK Biobank via the Complex-Traits Genetics Virtual Lab (https://genoma.io/). Complex-Traits Genetics Virtual Lab is a complementary and open-source program, which combines available GWAS datasets to facilitate genetic correlation estimation of complex characteristics using LDSC. The results were corrected for multiple testing using the Bonferroni method.

Phenome-wide association analysis (PheWAS)

We conducted a Phenome-Wide Association Analysis (PheWAS) to explore associations between significant SNP associations and their corresponding genes with other traits with two objectives: (1), aiming to validate GWAS results by confirming the associations we identified with pain phenotypes; and (2) identify novel relationships between significant genetic variants associated with neck or shoulder pain and other phenotypes (focusing on psychiatric phenotypes such as morningness, schizophrenia defined by ATLAS

https://atlas.ctglab.nl/PheWAS [7]). This analysis involved generating PheWAS plots using an extensive dataset of 4,756 GWAS summary statistics available on the GWAS ATLAS platform [7]. For this PheWAS, only SNPs with associations whose *p* values were lower than 0.05 were considered and adjustment for multiple comparisons was carried out using the Bonferroni correction method.

Mendelian Randomization analysis of neck or shoulder pain and associated traits

To investigate potential causal relationships between neck or shoulder pain and other traits, we performed a series of Mendelian Randomization (MR) analyses. We explored the genetic causality between neck or shoulder pain and three traits: schizophrenia [5], frequency of tiredness/lethargy in the past two weeks, and neuroticism [3]. All MR analyses were conducted using the TwoSampleMR tool and the data were sourced from publicly available IEU datasets with detailed dataset identifiers and sources provided in Supplementary Table 7. The results indicate significant causal relationships between neck or shoulder pain and all three traits. However, the causal relationship between schizophrenia and neck or shoulder pain could not be analyzed due to a lack of significant SNPs for matching. Detailed results are provided in supplementary table 8-12, including relevant figures in supplementary figures 10-14.

References:

1. Bulik-Sullivan BK, Loh P-R, Finucane HK, Ripke S, Yang J, Patterson N, Daly MJ, Price AL, Neale BM: LD Score regression distinguishes confounding from polygenicity in genome-wide association studies. Nat Genet Nature Publishing Group; 47:291–5, 2015.

2. Li G, Sun T, Chang H, Cai L, Hong P, Zhou Q: Chromatin Interaction Analysis with

Updated ChIA-PET Tool (V3). Genes (Basel) 10:554, 2019.

3. Loh P-R, Kichaev G, Gazal S, Schoech AP, Price AL: Mixed-model association for biobank-scale datasets. Nat Genet Nature Publishing Group; 50:906–8, 2018.

4. Pruim RJ, Welch RP, Sanna S, Teslovich TM, Chines PS, Gliedt TP, Boehnke M, Abecasis GR, Willer CJ: LocusZoom: regional visualization of genome-wide association scan results. Bioinformatics 26:2336–7, 2010.

5. Trubetskoy V, Pardiñas AF, Qi T, Panagiotaropoulou G, Awasthi S, Bigdeli TB, Bryois J, Chen C-Y, Dennison CA, Hall LS, Lam M, Watanabe K, Frei O, Ge T, Harwood JC, Koopmans F, Magnusson S, Richards AL, Sidorenko J, Wu Y, Zeng J, Grove J, Kim M, Li Z, Voloudakis G, Zhang W, Adams M, Agartz I, Atkinson EG, Agerbo E, Al Eissa M, Albus M, Alexander M, Alizadeh BZ, Alptekin K, Als TD, Amin F, Arolt V, Arrojo M, Athanasiu L, Azevedo MH, Bacanu SA, Bass NJ, Begemann M, Belliveau RA, Bene J, Benyamin B, Bergen SE, Blasi G, Bobes J, Bonassi S, Braun A, Bressan RA, Bromet EJ, Bruggeman R, Buckley PF, Buckner RL, Bybjerg-Grauholm J, Cahn W, Cairns MJ, Calkins ME, Carr VJ, Castle D, Catts SV, Chambert KD, Chan RCK, Chaumette B, Cheng W, Cheung EFC, Chong SA, Cohen D, Consoli A, Cordeiro Q, Costas J, Curtis C, Davidson M, Davis KL, de Haan L, Degenhardt F, DeLisi LE, Demontis D, Dickerson F, Dikeos D, Dinan T, Djurovic S, Duan J, Ducci G, Dudbridge F, Eriksson JG, Fañanás L, Faraone SV, Fiorentino A, Forstner A, Frank J, Freimer NB, Fromer M, Frustaci A, Gadelha A, Genovese G, et al.: Mapping genomic loci implicates genes and synaptic biology in schizophrenia. Nature Nature Publishing Group; 604:502–8, 2022.

6. Võsa U, Claringbould A, Westra H-J, Bonder MJ, Deelen P, Zeng B, Kirsten H, Saha A, Kreuzhuber R, Yazar S, Brugge H, Oelen R, de Vries DH, van der Wijst MGP, Kasela S, Pervjakova N, Alves I, Favé M-J, Agbessi M, Christiansen MW, Jansen R, Seppälä I, Tong L, Teumer A, Schramm K, Hemani G, Verlouw J, Yaghootkar H, Sönmez Flitman R, Brown A, Kukushkina V, Kalnapenkis A, Rüeger S, Porcu E, Kronberg J, Kettunen J, Lee B, Zhang F, Qi T, Hernandez JA, Arindrarto W, Beutner F, Dmitrieva J, Elansary M, Fairfax BP, Georges M, Heijmans BT, Hewitt AW, Kähönen M, Kim Y, Knight JC, Kovacs P, Krohn K, Li S, Loeffler M, Marigorta UM, Mei H, Momozawa Y, Müller-Nurasyid M, Nauck M, Nivard MG, Penninx BWJH, Pritchard JK, Raitakari OT, Rotzschke O, Slagboom EP, Stehouwer CDA, Stumvoll M, Sullivan P, ’t Hoen PAC, Thiery J, Tönjes A, van Dongen J, van Iterson M, Veldink JH, Völker U, Warmerdam R, Wijmenga C, Swertz M, Andiappan A, Montgomery GW, Ripatti S, Perola M, Kutalik Z, Dermitzakis E, Bergmann S, Frayling T, van Meurs J, Prokisch H, Ahsan H, Pierce BL, Lehtimäki T, Boomsma DI, Psaty BM, Gharib SA, Awadalla P, Milani L, Ouwehand WH, Downes K, et al.: Large-scale cis- and trans-eQTL analyses identify thousands of genetic loci and polygenic scores that regulate blood gene expression. Nat Genet Nature Publishing Group; 53:1300–10, 2021.

7. Watanabe K, Stringer S, Frei O, Umićević Mirkov M, de Leeuw C, Polderman TJC, van der Sluis S, Andreassen OA, Neale BM, Posthuma D: A global overview of pleiotropy and genetic architecture in complex traits. Nat Genet 51:1339–48, 2019.

8. Watanabe K, Taskesen E, van Bochoven A, Posthuma D: Functional mapping and annotation of genetic associations with FUMA. Nat Commun Nature Publishing Group; 8:1826, 2017.

9. Yang J, Lee SH, Goddard ME, Visscher PM: GCTA: A Tool for Genome-wide Complex Trait Analysis. Am J Hum Genet 88:76–82, 2011.

Supplementary Figures

Figure S1 The regional plots of loci in the *SLC38A8* and *FOXP2* regions from the primary GWAS.

Figure S2 Q-Q plot of observed versus expected *p* values from the primary GWAS.

Figure S3 Manhattan plot of gene-based associations computed by MAGMA for neck or shoulder pain.

Figure S4 Heatmaps of gene expression across 54 specific and 30 general tissue types from GTEx v8.

Figure S5 Circos plot of chromatin interactions and eQTLs associated with genomic risk loci.

Figure S6 Phenome-wide associations with significant SNPs for neck or shoulder pain.

Figure S7 Phenome-wide associations with significant genes for neck or shoulder pain.

Figure S8 GWAS for acute neck or shoulder pain

Figure S9 CaTS power calculator to estimate the statistical power

Figure S10 Mendelian Randomization analysis of neck or shoulder pain and associated traits

Figure S11 MR results of frequency of tiredness / lethargy in last 2 weeks to neck

or shoulder pain

Figure S12 MR results of neck or shoulder pain to frequency of tiredness / lethargy in last 2 weeks

Figure S13. MR results of neuroticism to neck or shoulder pain

Figure S14 MR results of neck or shoulder pain to neuroticism

Figure S15 4-Way venn diagram of significant SNPs across neck or shoulder, back, hip, and knee pain GWAS

Figure S16 3-Way and 4-Way venn diagrams of GWAS credible sets

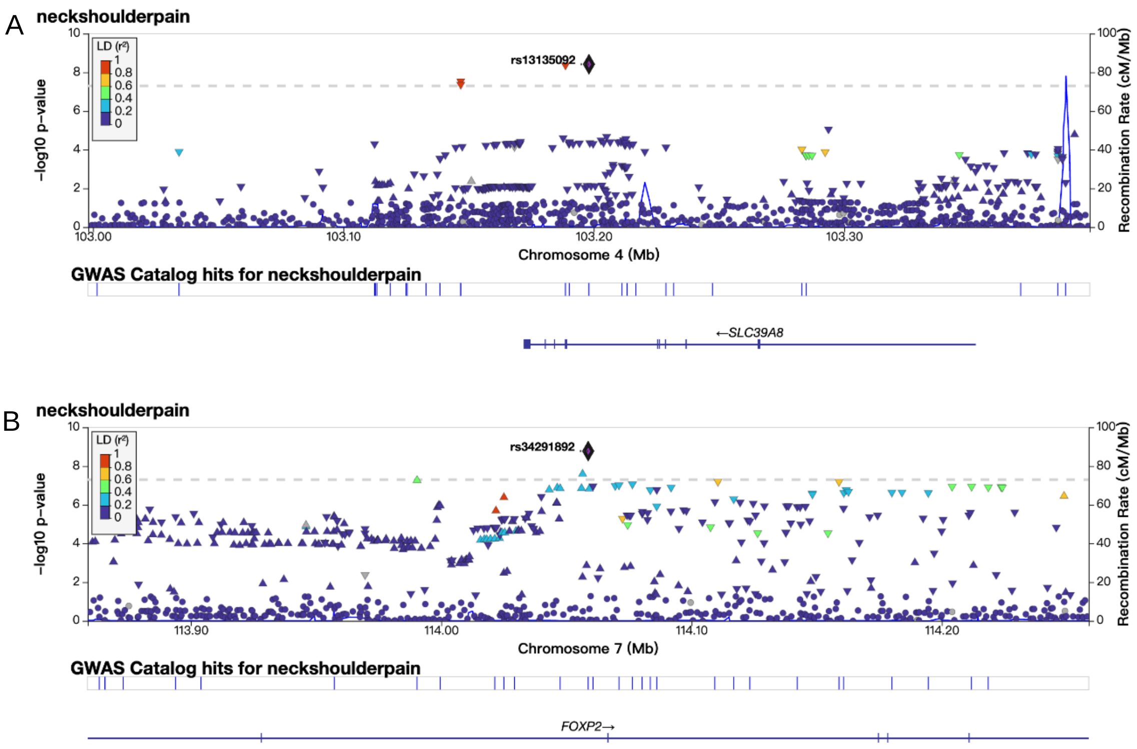

Figure S1 The regional plots of loci in the *SLC38A8* and *FOXP2* regions. Panel A shows the regional plot for the *SLC39A8* locus with lead SNP rs13135092 SNP marked in purple, and Panel B displays the regional plot for the *FOXP2* locus with the lead SNP rs34291892 SNP marked in purple. Each plot illustrates SNP associations in the respective regions from the primary GWAS analysis. The x-axis represents the genomic position in Mb, while the y-axis displays the association strength of each SNP as –log^{10} *p* value.

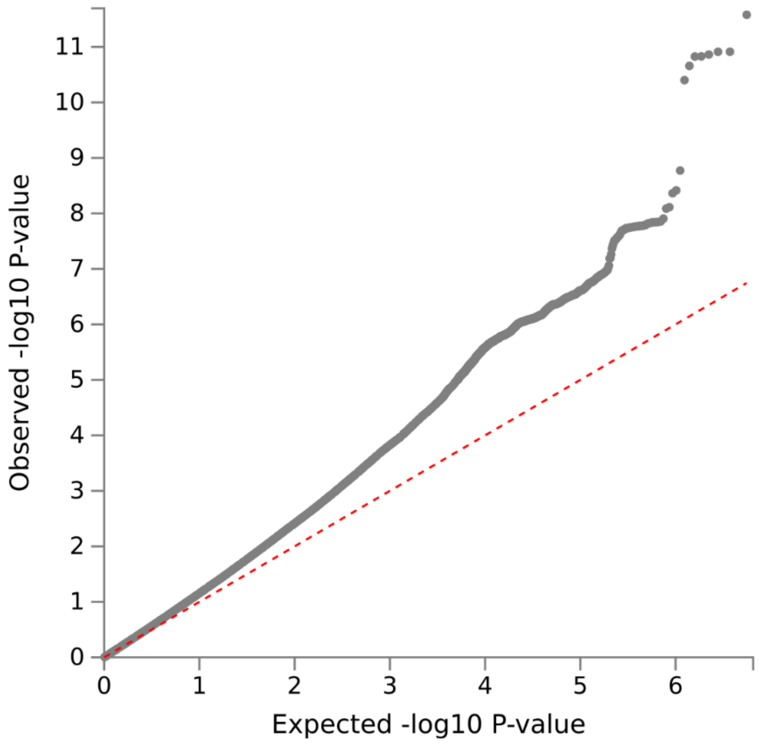

Figure S2 Q-Q plot of observed versus expected *p* values from the primary GWAS.

The Q-Q plot displays the observed versus expected *p* values from the primary GWAS. SNPs with *p* values ≤ 1x 10^{-5} are included in the plot, and overlapping data points are omitted for clarity. The x-axis represents the expected -log10(*p*) values under the null hypothesis, while the y-axis shows the observed -log10(*p*) values. Points deviating from the diagonal line indicate associations stronger than expected.

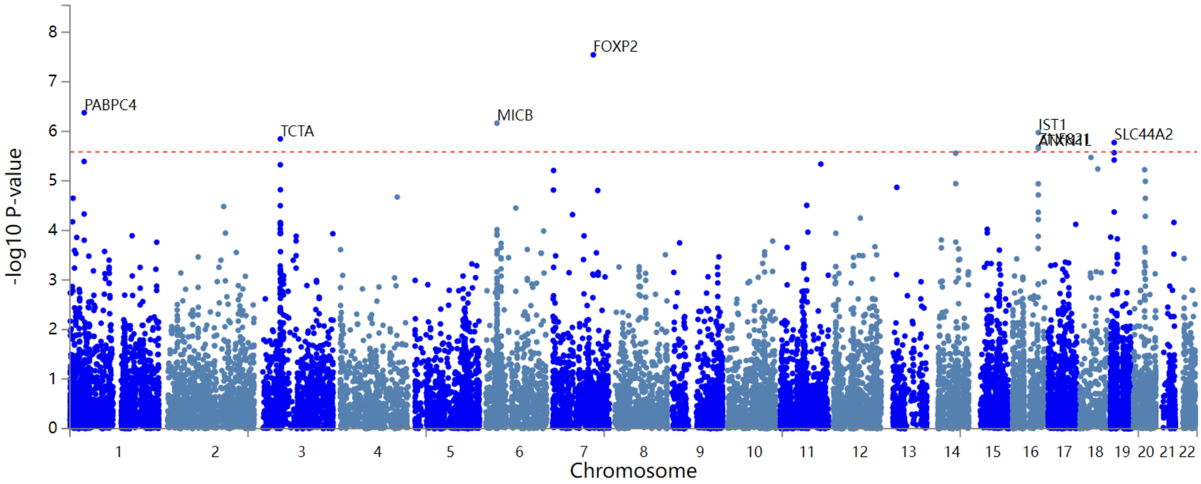

Figure S3 Manhattan plot of gene-based associations for neck or shoulder pain. The Manhattan plot displays the results of a gene-based test computed by MAGMA using GWAS summary statistics. Each dot represents a protein-coding gene mapped from the input SNPs (N = 19,203 genes). The y-axis shows the $-\log^{10}$(p value) of gene-based associations, while the x-axis represents genomic positions across chromosomes. The red dashed line denotes the genome-wide significance threshold (p = 0.05/19,203 = 2.604 × 10^{-6}). Genes that surpass this threshold are labeled in the plot, indicating their significant association with neck or shoulder pain.

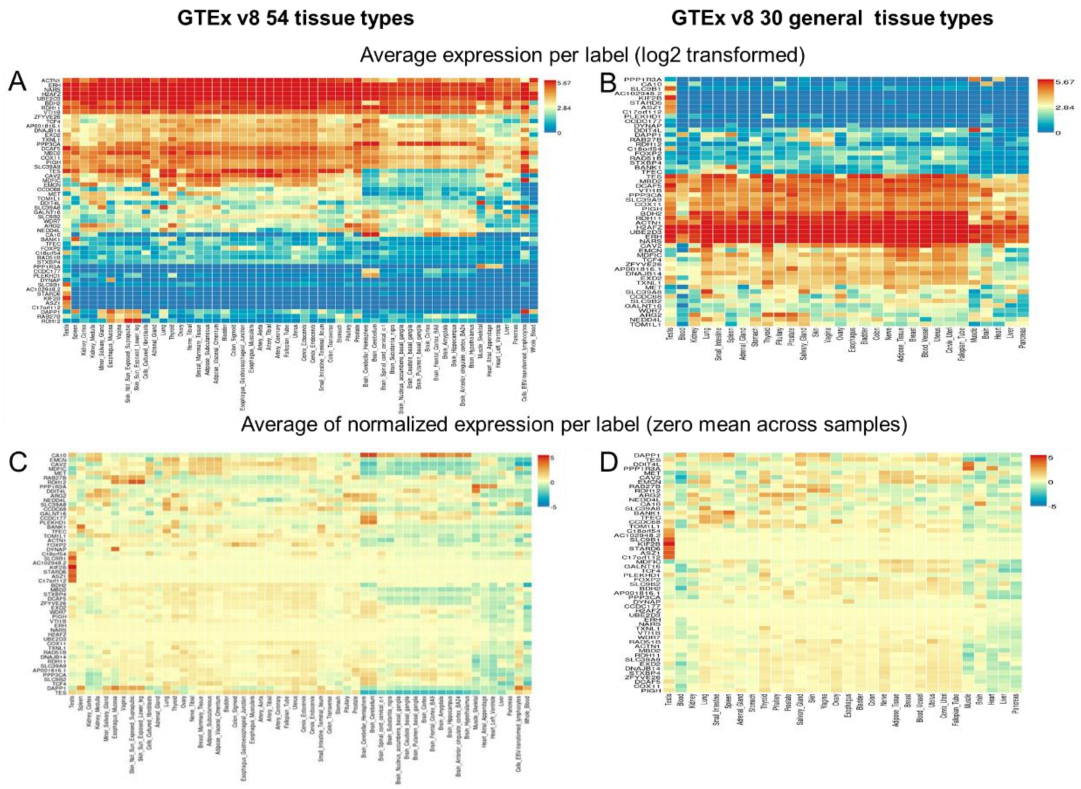

Figure S4 Heatmaps of gene expression across 54 specific and 30 general tissue types from GTEx v8. The four heatmaps visualize gene expression values for 54 specific tissue types and 30 general tissue types from GTEx v8, with genes represented as rows and tissues as columns. Panel A shows the average expression per label for 54 specific tissue types (log2 transformed). Panel B displays the average expression per label for 30 general tissue types (log2 transformed). Panel C represents the average of normalized expression per label (zero mean across samples) for 54 specific tissue types. Panel D shows the average of normalized expression per label (zero mean across samples) for 30 general tissue types. Red cells indicate higher expression relative to other tissues and genes, while blue cells indicate lower expression.

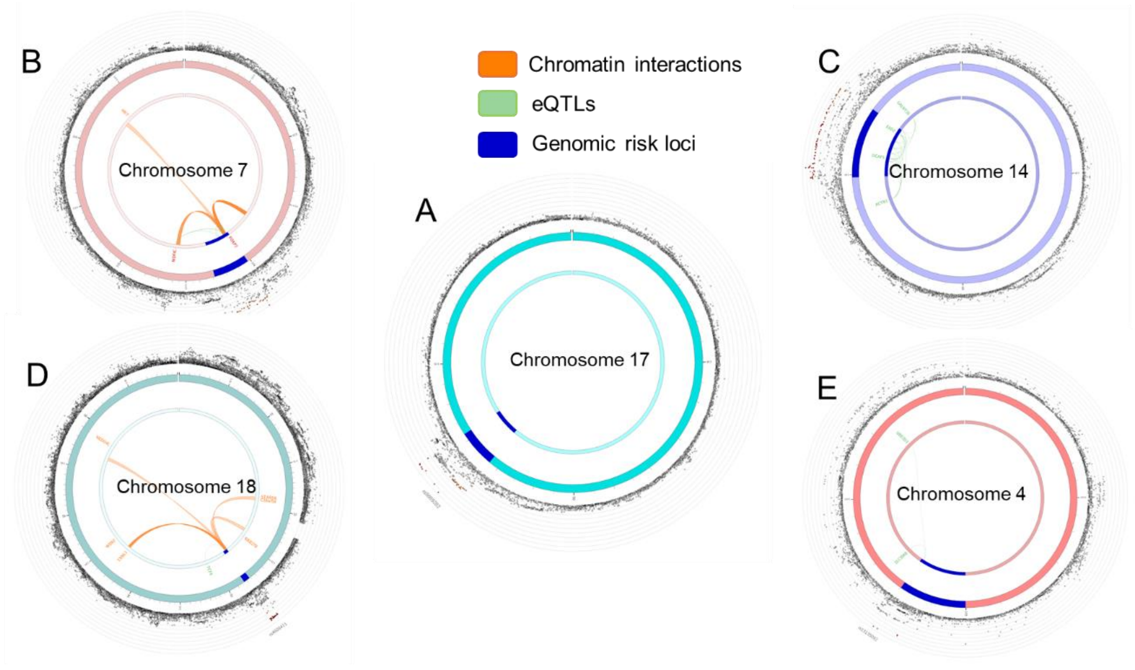

Figure S5 Circos plot of chromatin interactions and eQTLs associated with genomic risk loci. The Circos plot displays chromatin interactions and eQTL associated with genomic risk loci. The outermost layer shows a Manhattan plot with loci meeting the significance threshold of $p < 0.05$. Each SNP in a genomic risk locus is color-coded according to its maximum LD, measured by r^2 with an independent significant SNP in the locus: red ($r^2 > 0.8$), orange ($r^2 > 0.6$), green ($r^2 > 0.4$), and blue ($r^2 > 0.2$). The middle layer marks genomic risk loci (with lead SNP $p < 5 \times 10^{-8}$) in blue. The innermost layer highlights eQTLs (green) and/or chromatin interactions (orange). Panels A to E show specific top SNPs on chromosomes 17, 7, 14,18 and 4, respectively.

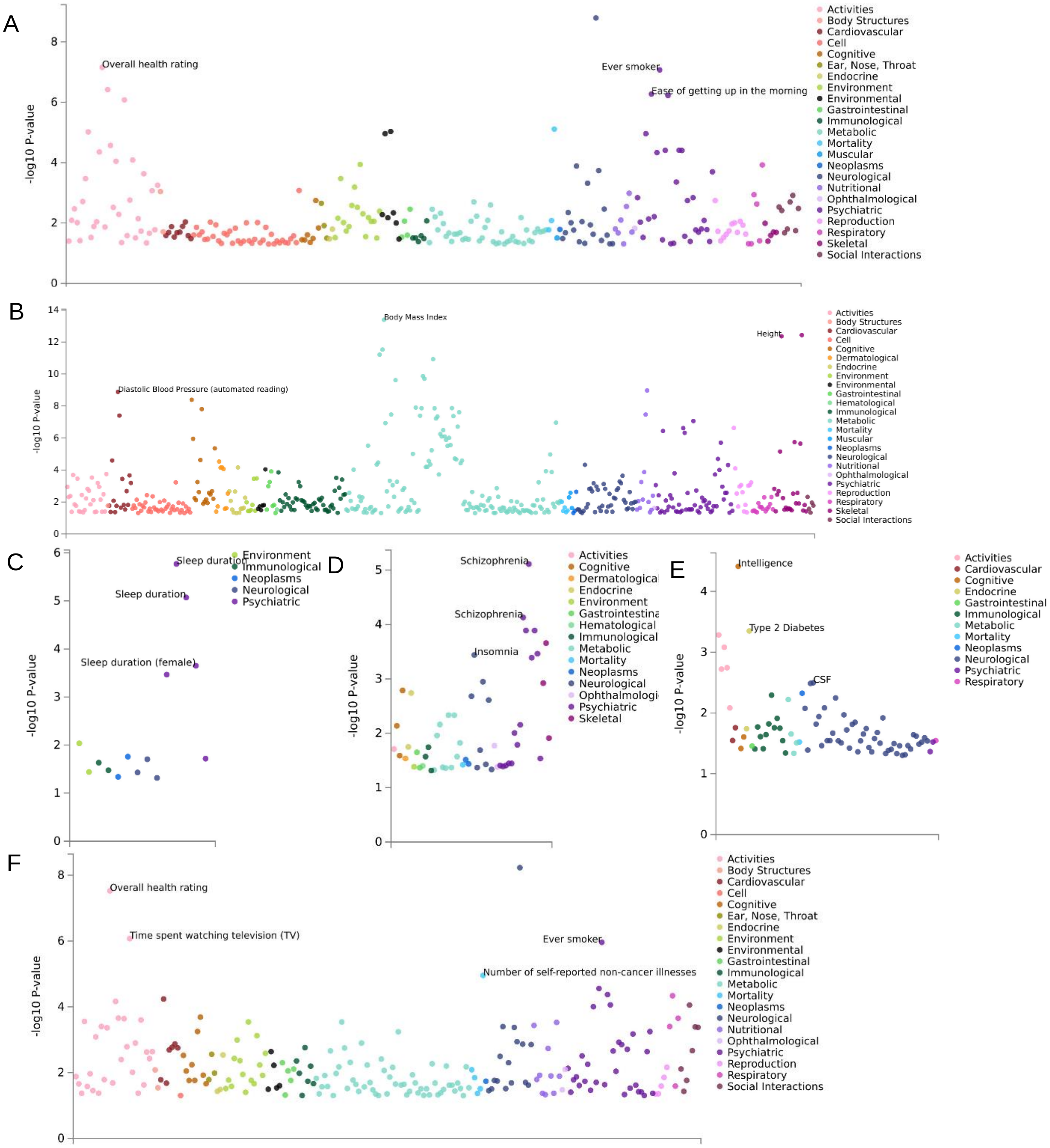

Figure S6 Phenome-wide associations with significant SNPs for neck or shoulder pain. This plot illustrates the phenome-wide associations of significant SNPs linked to neck or shoulder pain across various phenotypic categories. Each panel represents the associations for a specific SNP: Panel A shows results for rs9889282, Panel B for rs34291892, Panel C for rs13135092, Panel D for rs4608411, Panel E for rs370565192, and Panel F for rs12951067. Each dot represents a phenotype association, with the x-axis

showing phenotype categories and the y-axis displaying the -log[10]*p* value) for each association. Phenotypes are color-coded according to broader phenotype categories. Significant associations are labeled for clarity.

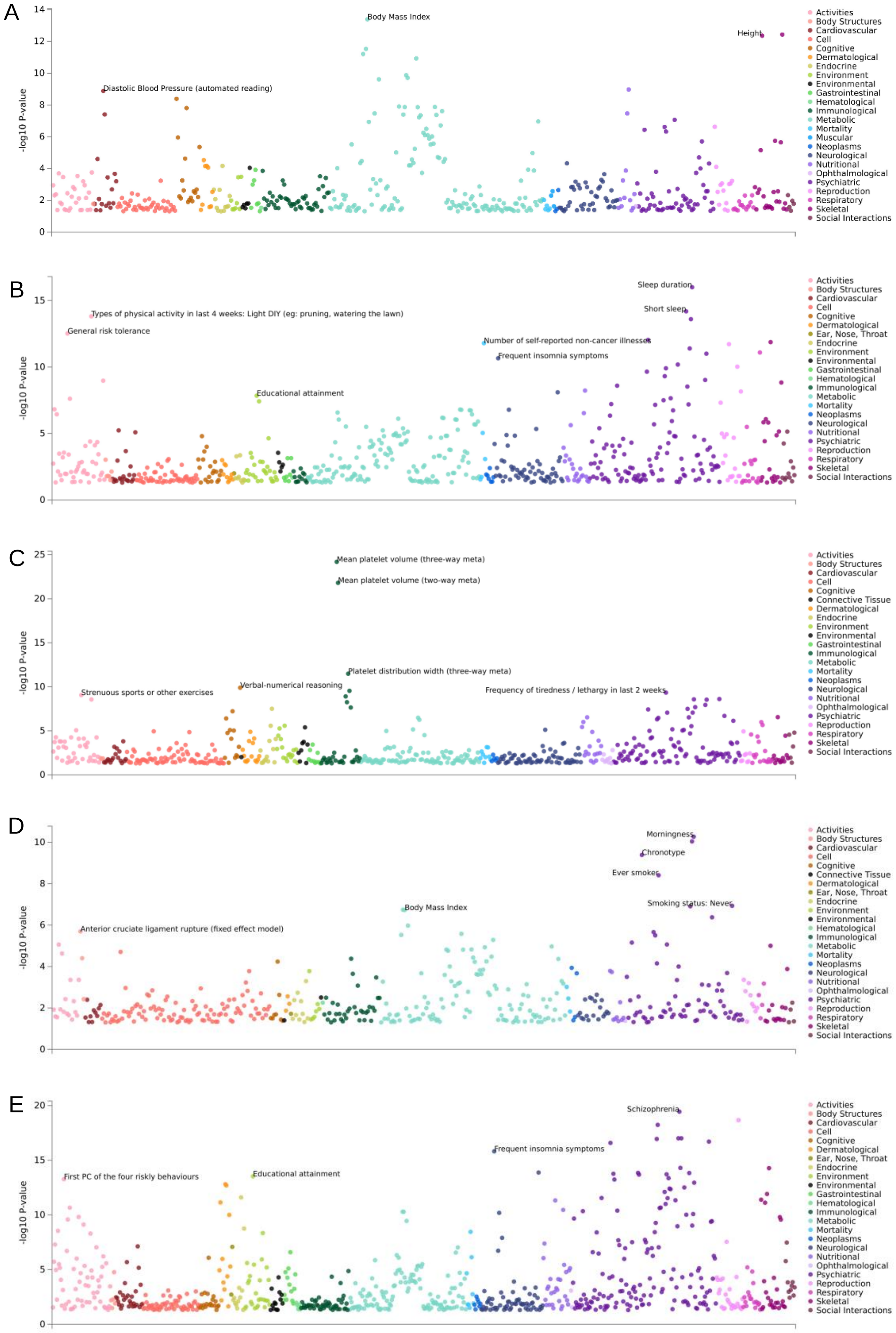

Figure S7 Phenome-wide associations with significant genes for neck or shoulder

pain. This plot illustrates the phenome-wide associations of significant genes linked to neck or shoulder pain across various phenotypic categories. Each panel represents the associations for a gene: Panel A shows results for *SLC39A8*, Panel B for *FOXP2*, Panel C for *DCF5*, Panel D for *CA10*, and Panel E for *TCF4*. Each dot represents a phenotype association, with the x-axis showing phenotype categories and the y-axis displaying the -log^{10}*p* value) for each association. Phenotypes are color-coded according to broader phenotype categories. Significant associations are labeled for clarity.

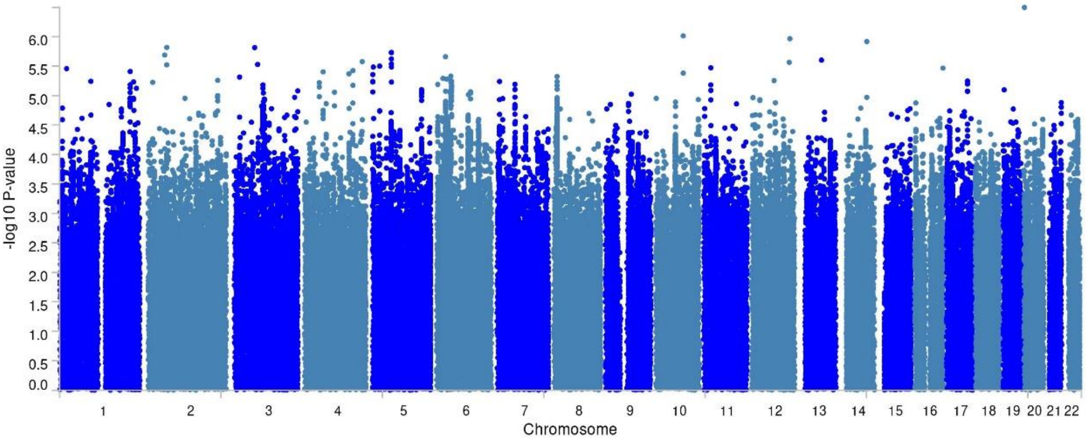

Figure S8 GWAS for acute neck or shoulder pain. The x-axis represents chromosome positions across the 22 autosomes, and the y-axis shows the $-\log^{10}$ *p* values of SNP associations. In response to the comment regarding the ambiguity of cases and controls in the UK Biobank cohort, we performed a separate GWAS for acute neck and shoulder pain by excluding individuals who answered "yes" in field 3404 (indicating chronic pain lasting 3 months or more). This analysis yielded no significant loci, likely due to a reduced sample size and the lower heritability of acute pain.

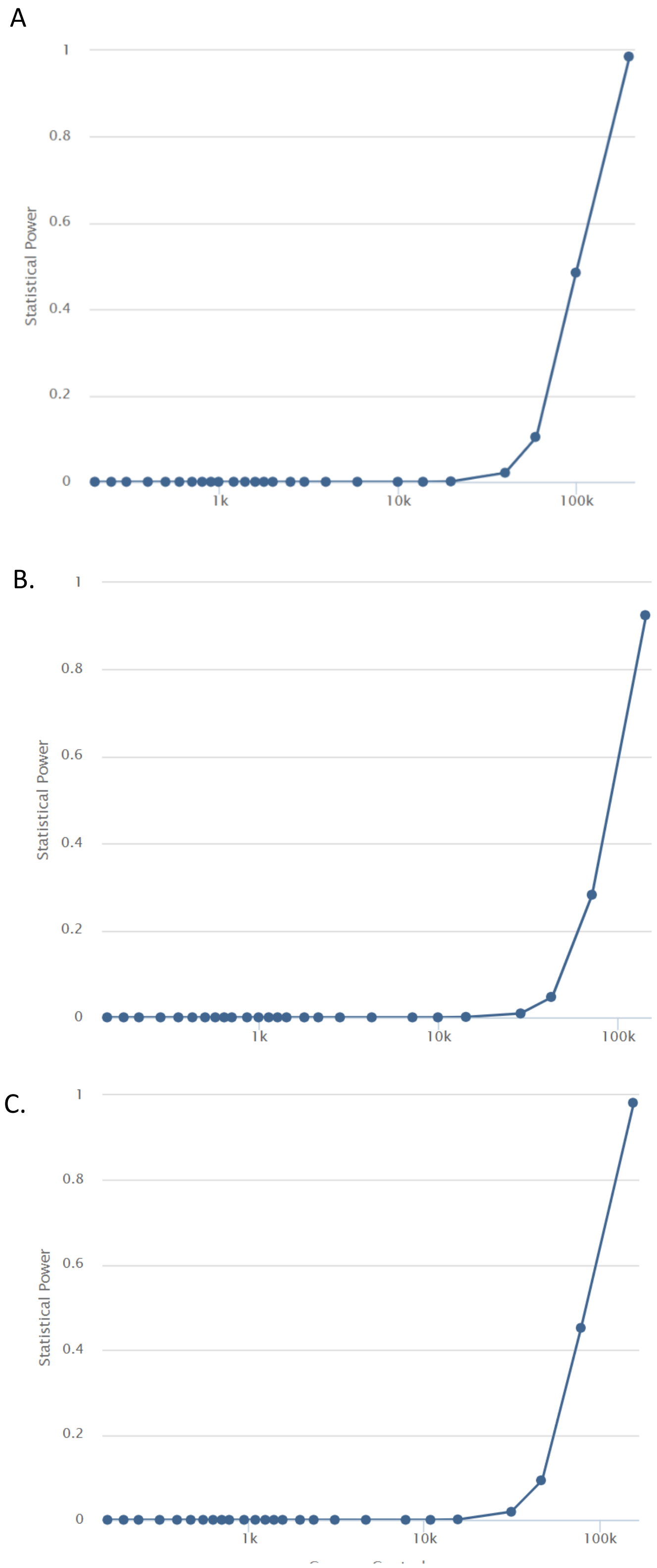

Figure S9 CaTS power calculator to estimate the statistical power. Panel A shows the power calculator result for the entire dataset. Panel B shows the power calculator result for the male cohort dataset. Panel C shows the power calculator result for the female cohort

dataset. We used the CaTS power calculator (**https://csg.sph.umich.edu/abecasis/gas_power_calculator/index.html**) to estimate statistical power for neck or shoulder pain. Assuming a significance level of 5 x 10^{-8}, a disease prevalence of 0.032, a disease allele frequency of 0.25 and a genotype relative risk of 1.06, the overall GWAS with 98,652 cases and 331,541 controls achieves an expected power of 99.8%. Stratified analysis by sex using the same parameters shows that for males (42,869 cases and 153,827 controls), the study achieves a power of 83.2%, while for females (55,783 cases and 177,714 controls), the power is 94.1%. Despite the smaller genotype relative risk of 1.06, the large overall sample size ensures sufficient power to detect contributing SNPs. The stratified analysis highlights that the power is slightly lower in males due to the smaller sample size but remains robust across both sexes.

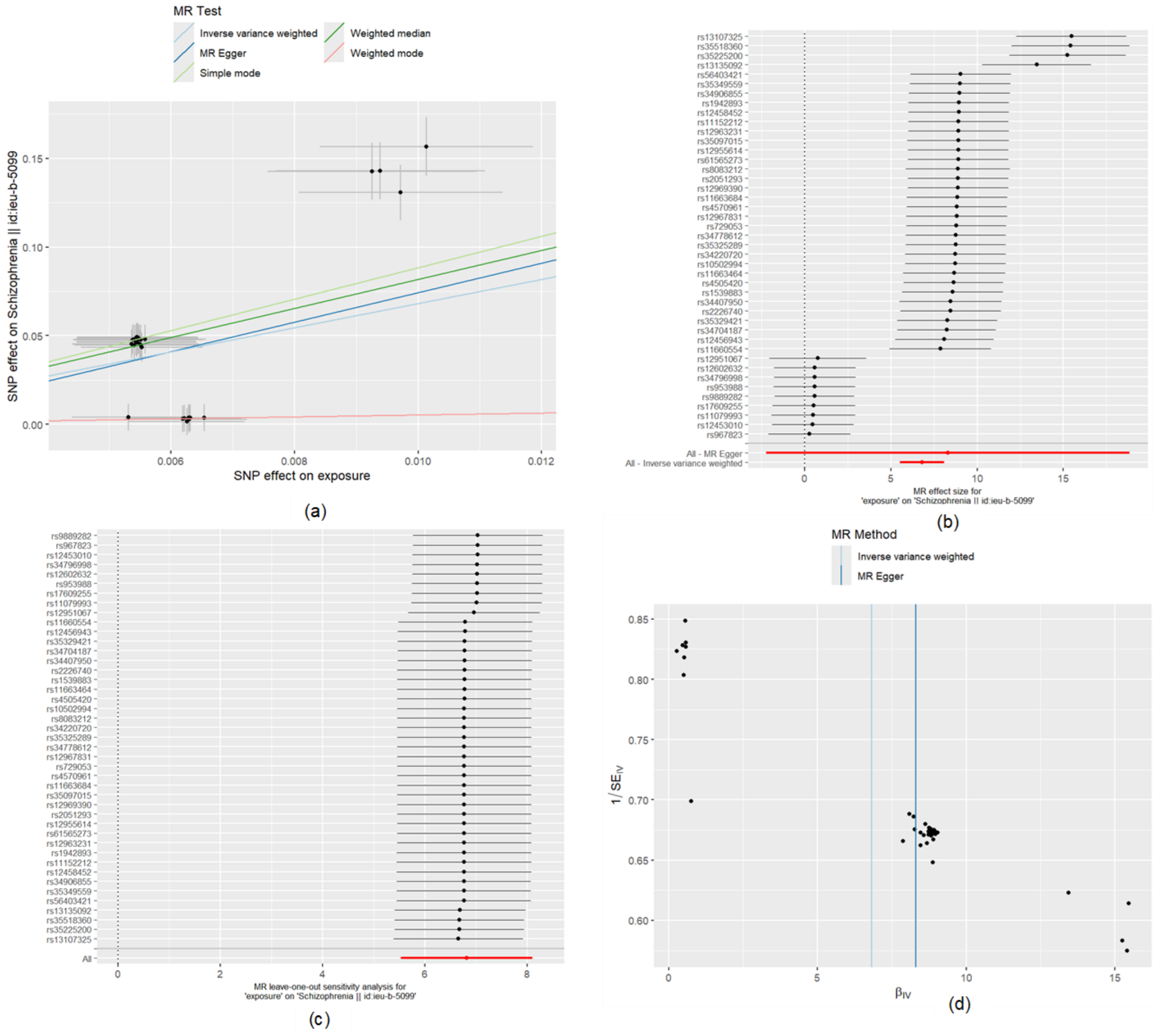

Figure S10 MR results of neck or shoulder pain to schizophrenia. Panel A shows a scatter plot of SNP effects on the exposure (X-axis) and their effects on the outcome (Y-axis). Panel B presents a forest plot of SNP effect estimates and directions for the exposure-outcome relationship. Panel C shows a forest plot assessing the impact of each SNP on the MR analysis, with sensitivity analysis by sequentially excluding SNPs to test result stability. Panel D illustrates a funnel plot assessing SNP heterogeneity. The detailed data on MR results of neck or shoulder pain to schizophrenia was in the Supplementary Table 8.

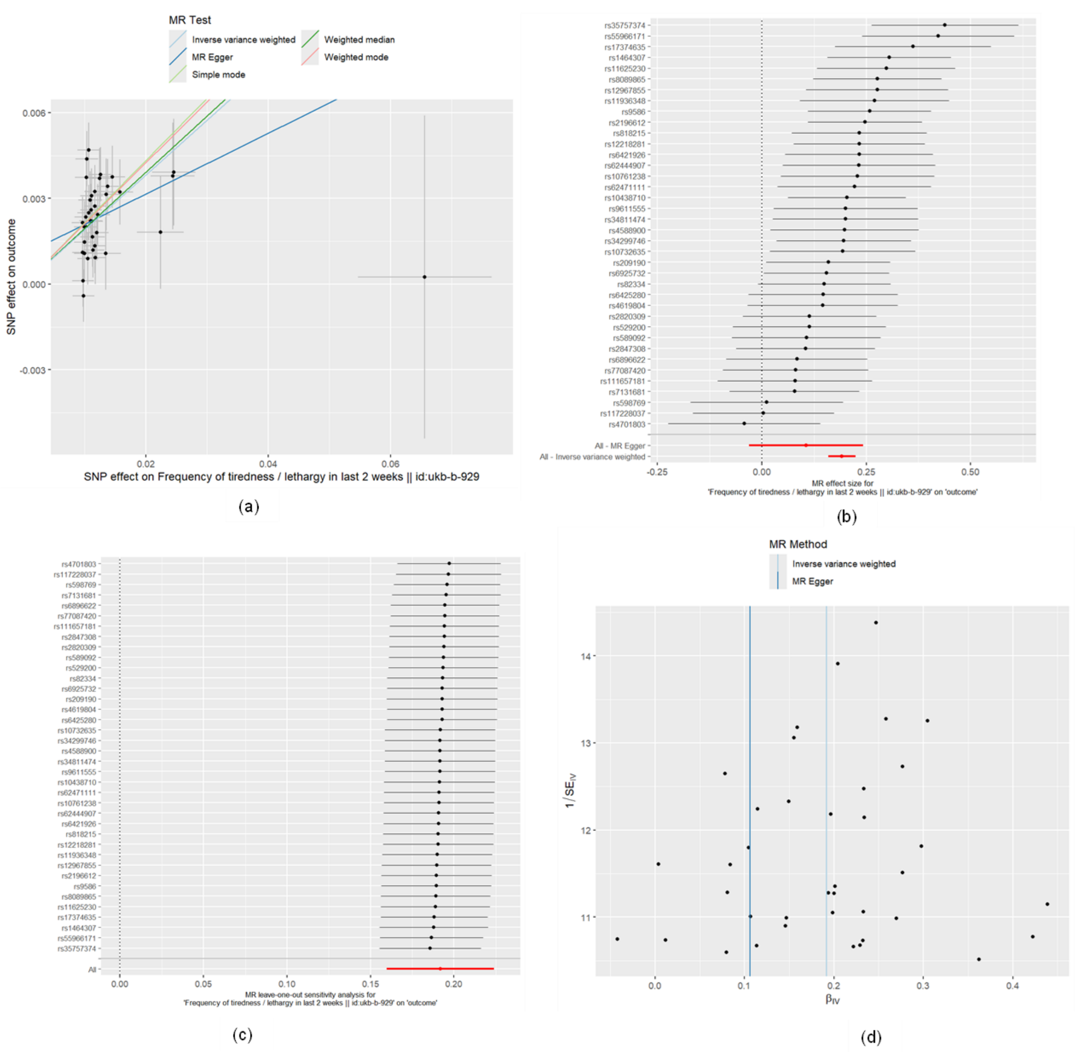

Figure S11 MR results of frequency of tiredness / lethargy in last 2 weeks to neck or shoulder pain. Panel A shows a scatter plot of SNP effects on the exposure (X-axis) and their effects on the outcome (Y-axis). Panel B presents a forest plot of SNP effect estimates and directions for the exposure-outcome relationship. Panel C shows a forest plot assessing the impact of each SNP on the MR analysis, with sensitivity analysis by sequentially excluding SNPs to test result stability. Panel D illustrates a funnel plot assessing SNP heterogeneity. The detailed data on MR results of tiredness / lethargy in last 2 weeks to neck or shoulder pain was in the Supplementary Table 9.

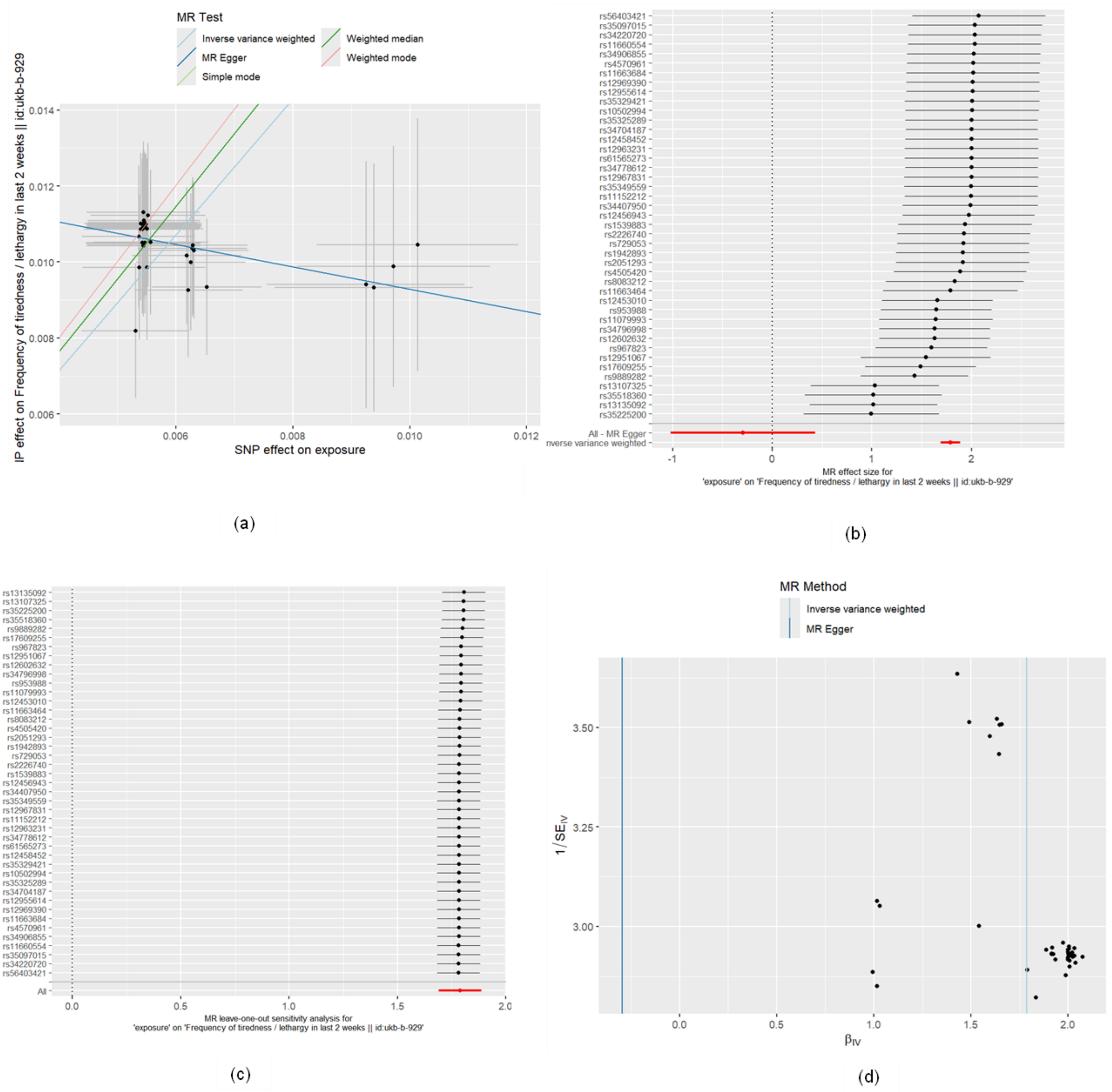

Figure S12 MR results of neck or shoulder pain to frequency of tiredness / lethargy in last 2 weeks. Panel A shows a scatter plot of SNP effects on the exposure (X-axis) and their effects on the outcome (Y-axis). Panel B presents a forest plot of SNP effect estimates and directions for the exposure-outcome relationship. Panel C shows a forest plot assessing the impact of each SNP on the MR analysis, with sensitivity analysis by sequentially excluding SNPs to test result stability. Panel D illustrates a funnel plot assessing SNP heterogeneity. The detailed data on MR results of neck or shoulder pain to frequency of tiredness / lethargy in last 2 weeks was in the Supplementary Table 10.

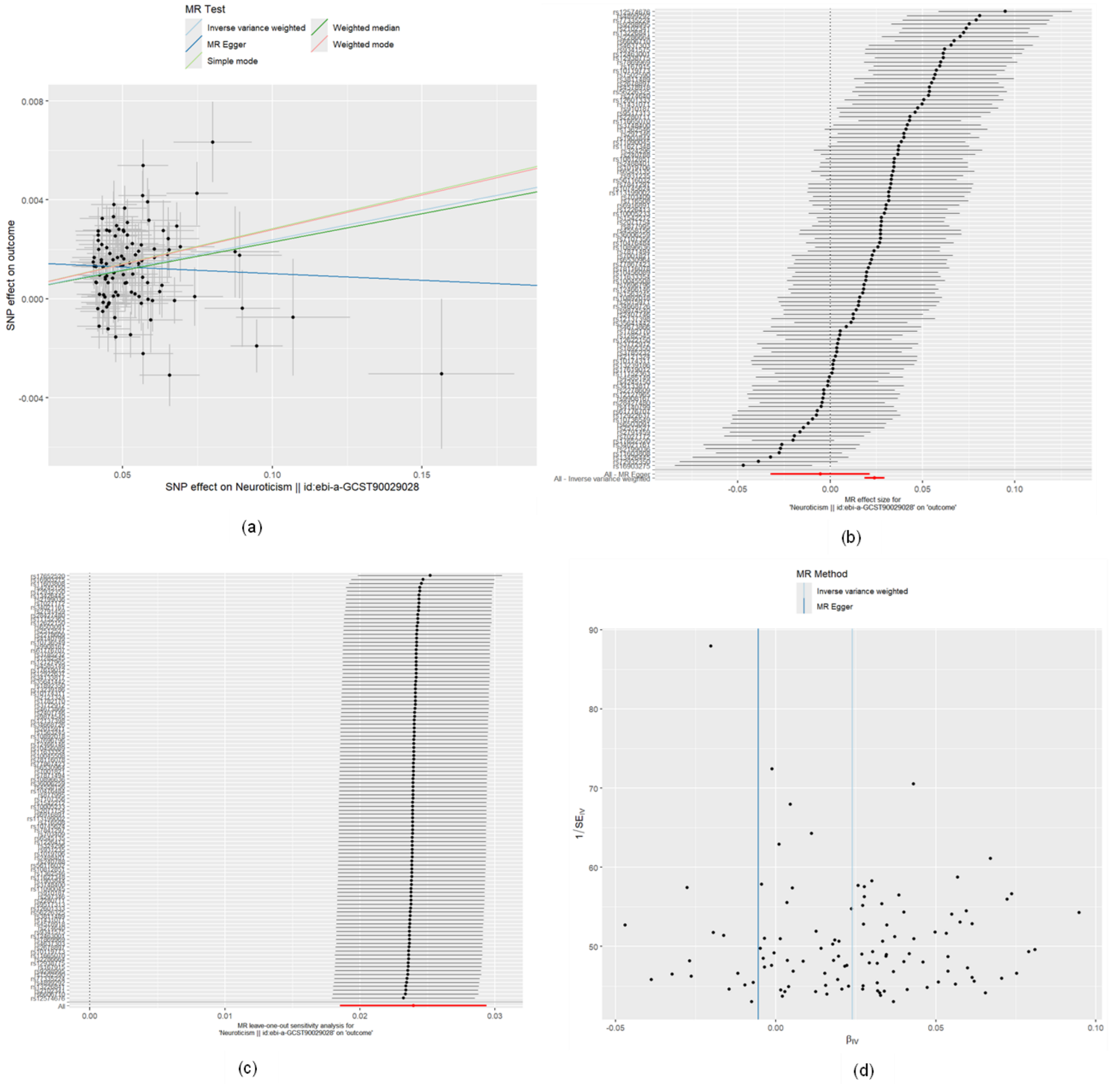

Figure S13. MR results of neuroticism to neck or shoulder pain. Panel A shows a scatter plot of SNP effects on the exposure (X-axis) and their effects on the outcome (Y-axis). Panel B presents a forest plot of SNP effect estimates and directions for the exposure-outcome relationship. Panel C shows a forest plot assessing the impact of each SNP on the MR analysis, with sensitivity analysis by sequentially excluding SNPs to test result stability. Panel D illustrates a funnel plot assessing SNP heterogeneity. The detailed data on MR results of neuroticism to neck or shoulder pain was in the Supplementary Table 11.

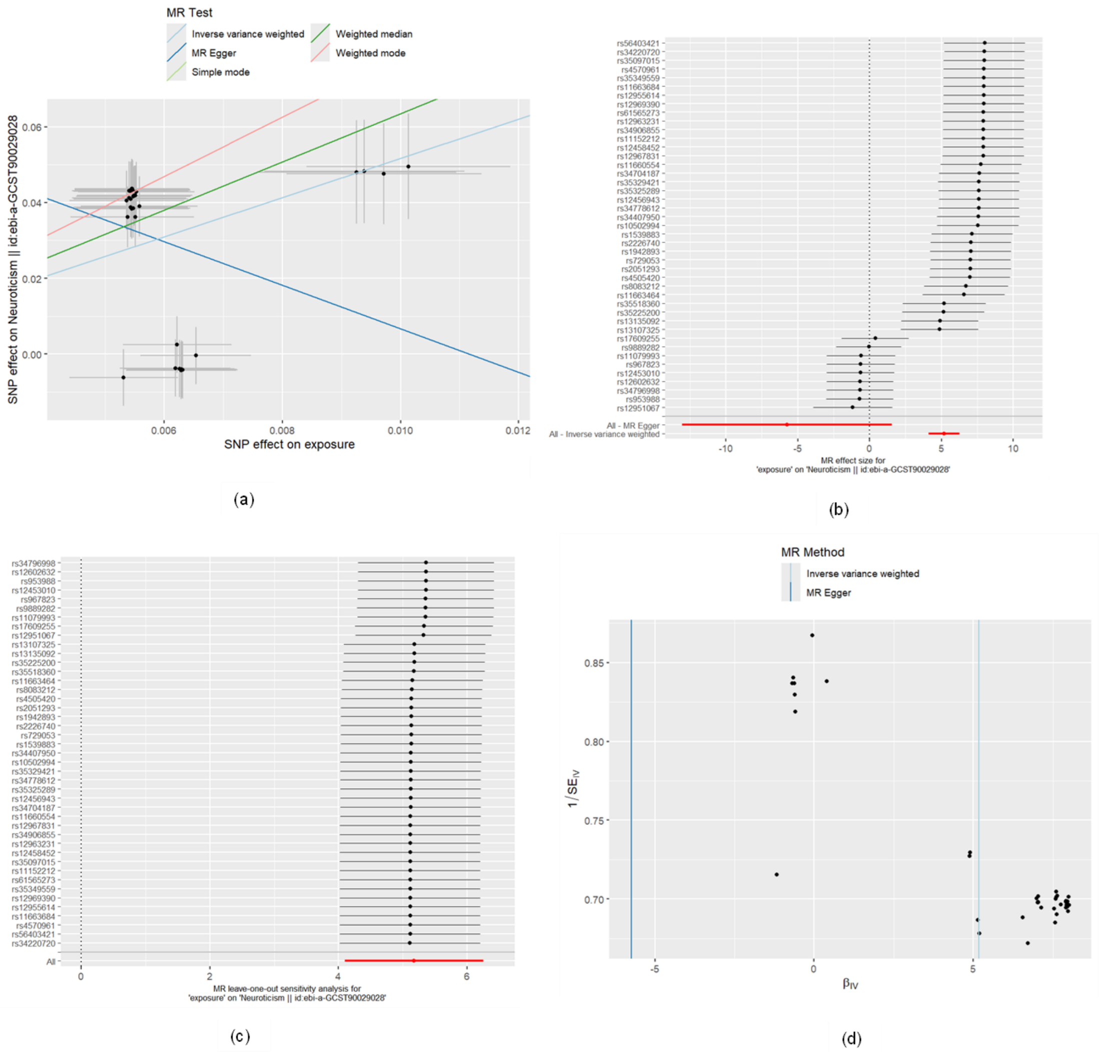

Figure S14 MR results of neck or shoulder pain to neuroticism. Panel A shows a scatter plot of SNP effects on the exposure (X-axis) and their effects on the outcome (Y-axis). Panel B presents a forest plot of SNP effect estimates and directions for the exposure-outcome relationship. Panel C shows a forest plot assessing the impact of each SNP on the MR analysis, with sensitivity analysis by sequentially excluding SNPs to test result stability. Panel D illustrates a funnel plot assessing SNP heterogeneity. The detailed data on MR results of neck or shoulder pain to neuroticism was in the Supplementary Table 12.

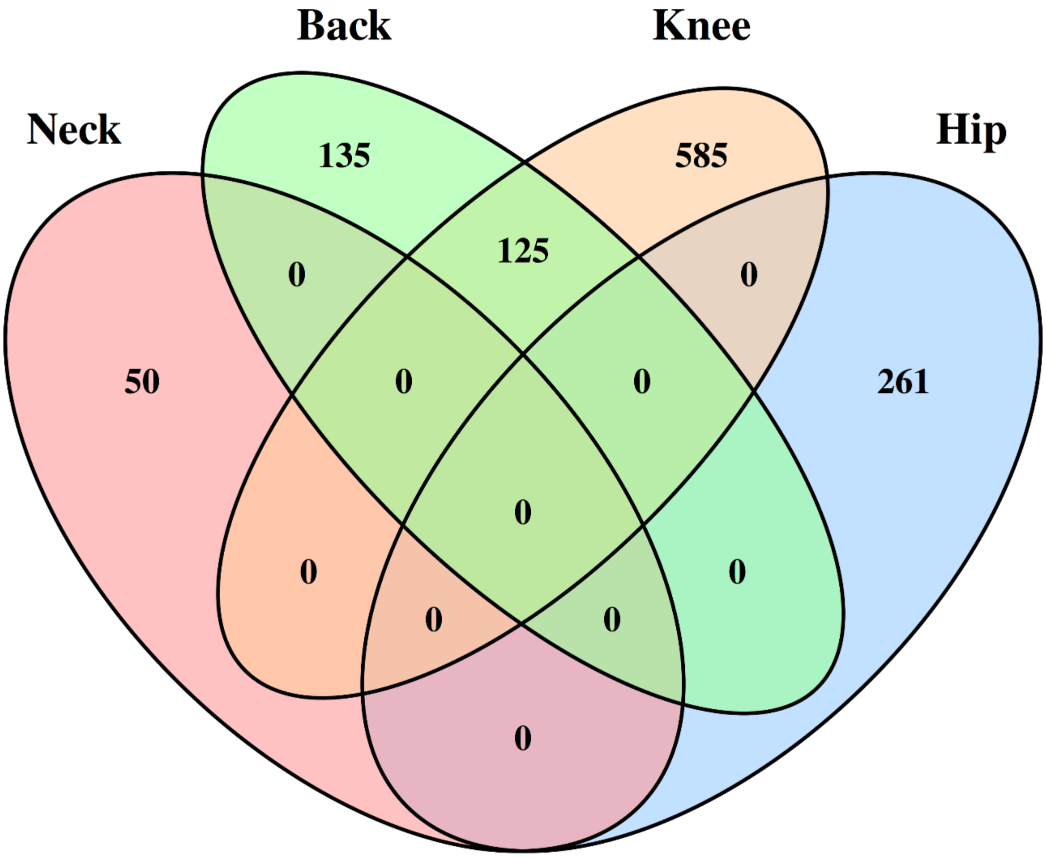

Figure S15 4-way venn diagram of significant SNPs across neck or shoulder, back, hip, and knee pain GWAS. This Venn diagram illustrates the overlap and uniqueness of significant SNPs identified in GWAS for neck or shoulder, back, hip, and knee pain. The pink area represents SNPs associated with neck/shoulder pain, the green area represents back pain, the blue area represents hip pain, and the orange area represents knee pain. The numbers indicate significant SNPs shared between or unique to each phenotype. The diagram shows that there is minimal overlap between the traits, with only a small number of significant SNPs shared between back and knee pain.

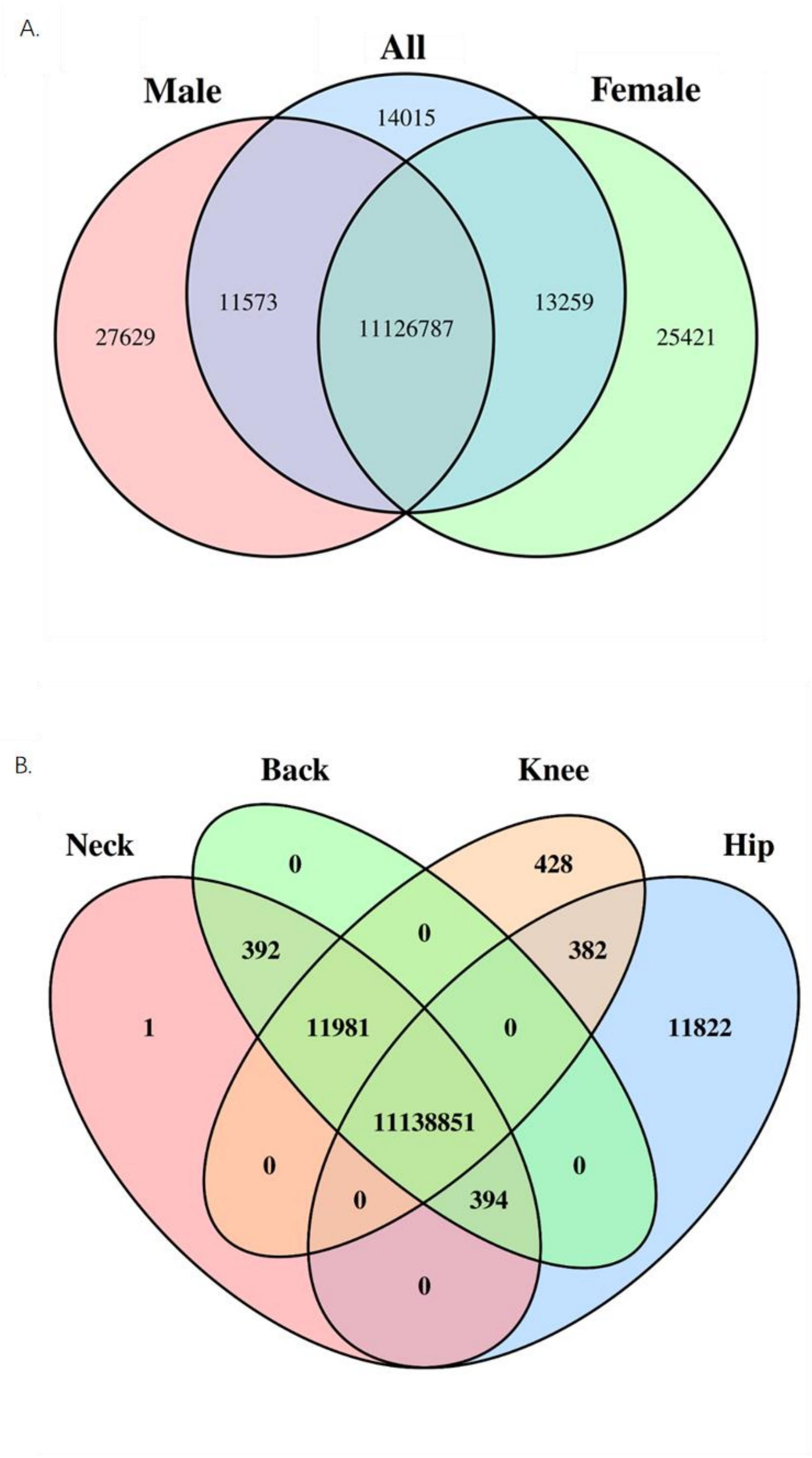

Figure S16 3-Way and 4-Way venn diagrams of GWAS credible sets. The Venn diagrams illustrate the shared and unique credible sets between the sex-combined, female, and male GWAS, as well as among multiple phenotypes. Panel A presents a 3-way Venn

diagram illustrating the overlap and uniqueness of credible sets identified in sex-combined (blue), male-specific (red), and female-specific (green) GWAS results. Panel B displays a 4-way Venn diagram showing the overlap and uniqueness of credible sets across neck (pink), back (green), knee (orange), and hip (blue) pain GWAS. The numbers represent the credible sets shared between or unique to each group or phenotype. The analyses were conducted using the prior probabilities calculated by PolyFun.

Supplementary tables

Table S1 A comprehensive list of all SNPS that are significantly associated in the primary GWAS

Table S2 The top ten gene sets identified in the gene-set analysis

Table S3 Significant cis-eQTL associations and mapped genes

Table S4 Genetic correlations between neck or shoulder pain and other phenotypes

Table S5 Phenotypic associations for SNPs (rs9889282, rs34291892, rs13135092, rs4608411, rs370565192, rs12951067) and genes (*CA10*, *SLC39A8*, *FOXP2*, *DCAF5*, *TCF4*).

Table S6 Results of the sex-difference meta-analysis using GWAMA

Table S7 Comprehensive details of IEU GWAS datasets for MR analyses

Table S8 MR results of neck or shoulder pain to schizophrenia

Table S9 MR results of frequency of tiredness / lethargy in last 2 weeks to neck or shoulder pain

Table S10 MR results of neck or shoulder pain to Frequency of tiredness / lethargy in last 2 weeks

Table S11 MR results of neuroticism to neck or shoulder pain

Table S12 MR results of neck or shoulder pain to neuroticism
